# Activation of epidermal growth factor receptor is required for *Chlamydia trachomatis* development

**DOI:** 10.1186/s12866-014-0277-4

**Published:** 2014-12-04

**Authors:** Achchhe L Patel, Xiaofei Chen, Scott T Wood, Elizabeth S Stuart, Kathleen F Arcaro, Doris P Molina, Snezana Petrovic, Cristina M Furdui, Allen W Tsang

**Affiliations:** Section on Molecular Medicine, Department of Internal Medicine, Wake Forest School of Medicine, Winston-Salem, NC 27109 USA; Department of Veterinary and Animal Science, University of Massachusetts, Amherst, MA 01003 USA; Department of Physiology and Pharmacology, Wake Forest School of Medicine, Winston-Salem, NC 27109 USA

## Abstract

**Background:**

*Chlamydia trachomatis* (*C. trachomatis*) is a clinically significant human pathogen and one of the leading causative agents of sexually transmitted diseases. As obligate intracellular bacteria, *C. trachomatis* has evolved strategies to redirect the host’s signaling and resources for its own survival and propagation. Despite the clinical notoriety of *Chlamydia* infections, the molecular interactions between *C. trachomatis* and its host cell proteins remain elusive.

**Results:**

In this study, we focused on the involvement of the host cell epidermal growth factor receptor (EGFR) in *C. trachomatis* attachment and development. A combination of molecular approaches, pharmacological agents and cell lines were used to demonstrate distinct functional requirements of EGFR in *C. trachomatis* infection. We show that *C. trachomatis* increases the phosphorylation of EGFR and of its downstream effectors PLCγ1, Akt and STAT5. While both EGFR and platelet-derived growth factor receptor-β (PDGFRβ) are partially involved in bacterial attachment to the host cell surface, it is only the knockdown of EGFR and not PDGFRβ that affects the formation of *C. trachomatis* inclusions in the host cells. Inhibition of EGFR results in small immature inclusions, and prevents *C. trachomatis*-induced intracellular calcium mobilization and the assembly of the characteristic F-actin ring at the inclusion periphery. By using complementary approaches, we demonstrate that the coordinated regulation of both calcium mobilization and F-actin assembly by EGFR are necessary for maturation of chlamydial inclusion within the host cells. A particularly important finding of this study is the co-localization of EGFR with the F-actin at the periphery of *C. trachomatis* inclusion where it may function to nucleate the assembly of signaling protein complexes for cytoskeletal remodeling required for *C. trachomatis* development.

**Conclusion:**

Cumulatively, the data reported here connect the function of EGFR to *C. trachomatis* attachment and development in the host cells, and this could lead to new venues for targeting *C. trachomatis* infections and associated diseases.

**Electronic supplementary material:**

The online version of this article (doi:10.1186/s12866-014-0277-4) contains supplementary material, which is available to authorized users.

## Author summary

*C. trachomatis* is one of the leading causative agents of sexually transmitted diseases. As an intracellular pathogen it has evolved strategies to redirect hosts’ signaling and resources for its own survival and propagation. The recruitment of tyrosine phosphorylated proteins at the site of entry in the host cell and the requirement of actin polymerization along the time course of infection are well documented. However, a function of receptor tyrosine kinases beyond the stages of attachment and entry in the host cell has never been reported. The studies presented here show that expression and phosphorylation of host cell epidermal growth factor receptor (EGFR) is required for *C. trachomatis* development*.* Most importantly, *C. trachomatis* can regulate the phosphorylation and intracellular localization of EGFR. Co-localization of EGFR with the F-actin at the periphery of *C. trachomatis* inclusion in the host cells is a particularly exciting and novel finding implicating EGFR in the regulation of actin polymerization around *C. trachomatis* inclusions. These studies open the opportunity to investigate key structural and functional elements in EGFR that are necessary for *C. trachomatis* development and which could lead to new therapies to advance the treatment of *C. trachomatis* infections and associated diseases.

## Background

*Chlamydia trachomatis* (*C. trachomatis*) is among the most common sexually transmitted pathogens in the US and contributes to many conditions, such as pelvic inflammatory disease [1,2], infertility [3], and others [4-6]. *C. trachomatis* has a small genome, ~1.0 Mb, and like viruses (e.g., HPV) depend on the host cell for survival [7-11]. The chlamydial life cycle exhibits two forms that are relevant to chlamydial pathology. The elementary body (EB) is a ‘spore-like’ infectious form, previously perceived as metabolically inert but recently shown to display maintenance level of metabolic activity [12,13]. Following internalization into the host cells, EBs initiate the inclusion formation and transform into metabolically active reticulate bodies (RBs), which then replicate within the inclusion. During the time course of RB replication, the early inclusions expand and fuse to form the early-mid inclusion, which then further expands into the mid-late inclusion. At this stage the RBs are converted back into EBs and are then released from the host cells through extrusion or cell lysis [14]. The process of *C. trachomatis* development from attachment/entry to extrusion/exit, is regulated by an arsenal of *C. trachomatis* and host cell proteins [15]. For example, several groups reported the recruitment of tyrosine-phosphorylated host cell proteins at the site of *C. trachomatis* entry into the host cell [16,17] and the requirement of actin polymerization along the time course of infection [18]. In accordance with this, previous studies have shown that *Chlamydia muridarum (C. muridarum*), a species closely related to *C. trachomatis*, induces activation of two host cell surface receptor tyrosine kinases: the fibroblast growth factor receptor (FGFR), and the platelet derived growth factor receptor β (PDGFRβ) [19,20]. FGFR and PDGFRβ have been proposed to be important for binding of the chlamydial EBs to the host cell. Elwell and co-workers have shown that PDGFRβ is phosphorylated upon *C. muridarum* infection and can function as a receptor for bacterial binding to the host cell. A function for PDGFR activation beyond this stage was not reported [19]. In an elegant study performed by Kim *et al*., it was shown that *C. muridarum* recruits FGF2 signaling to enhance infection and bacterial spread [20]. In this case, FGF2 acts as a bridging molecule between the EBs and the receptor that results in the activation of FGFR and bacterial uptake in the host cells.

The question therefore arises whether some of the other receptor tyrosine kinases play a similar function in the bacterial uptake or have functions that extend beyond this initial stage of bacterial infection. Of particular interest to our research is the epidermal growth factor receptor (EGFR), the activation and overexpression of which has been linked to malignant transformation and progression of a broad variety of cancers [21]. With respect to cancer, similar studies have shown that infection with *C. trachomatis* may contribute to malignant transformation as a co-factor with HPV or independent of HPV [22,23]. Moreover, Fischer *et al.* have shown in clinical studies an association between EGFR expression and *C. trachomatis* infection in women with intraepithelial neoplasia and with invasive carcinoma of the cervix [24]. The involvement of EGFR in chlamydial infection has been further shown in *Chlamydia pneumoniae (C. pneumoniae),* in which the protein Pmp21 binds to and activates EGFR to facilitate host cell entry [25]. A function of EGFR beyond entry was however not established. Increased activity of EGFR was also observed in a number of other infectious diseases. Zhang *et al*. have studied the function of host cell EGFR for *Pseudomonas aeruginosa* in which they show that, during infection, the activity of EGFR is enhanced followed by up-regulation of the downstream PI3K and Erk1/2 pathway [26]. Significant changes in the levels and activity of host signaling molecules like Akt, Erk1/2 and the downstream Bad protein during the developmental cycle of *Chlamydia* have also been reported [27-29]. Similarly, Swanson *et al.* have shown an enhanced *Neisseria gonorrhoeae*-induced activity of EGFR, which facilitated the gonococcal invasion [30].

EGFR signaling is also an important regulator of cytoskeleton remodeling in cells [31] and, over the years, several research groups have put forward the significance of host cytoskeletal rearrangement in the chlamydial development cycle [18,32,33]. Interestingly, EGFR has an F-actin binding domain (residues 985–996) [34], that is not present in PDGFRβ or FGFR. The functional consequence of the interaction between EGFR and the actin cytoskeleton is complex with recent evidence suggesting a role for the F-actin organization in EGFR dimer formation and polarized response to growth factor stimulation [35].

Altogether, the evidence brought about by the studies summarized above has prompted us to investigate the potential significance of host cell EGFR in *C. trachomatis* development and progression of *C. trachomatis*-associated diseases. We provide here the evidence identifying EGFR signaling as the first host cell receptor pathway required for *C. trachomatis* development within the host cell. Our data show: **a)** distinct functional requirements of EGFR *versus* PDGFR during *C. trachomatis* infection - we demonstrate that PDGFR is critical only at the step of bacterial attachment, and that knockdown of EGFR but not PDGFR impairs development of *C. trachomatis* inclusions within the host cell; **b)** infection with *C. trachomatis* increases phosphorylation of EGFR and of its downstream effectors PLCγ1, Akt and STAT5; **c) ***C. trachomatis* infection results in re-localization of EGFR at the periphery of *C. trachomatis* inclusion inside the host cell; and **d)** inhibition of EGFR results in the formation of a diffuse assembly of F-actin at the periphery of incompletely developed inclusions. Co-localization of EGFR with F-actin at the periphery of *C. trachomatis* inclusion is a particularly exciting and novel finding implicating EGFR in the regulation of actin polymerization around *C. trachomatis* inclusions.

## Results

### *C. trachomatis* induces EGFR phosphorylation and activation of EGFR signaling pathways

**Figure 1:**
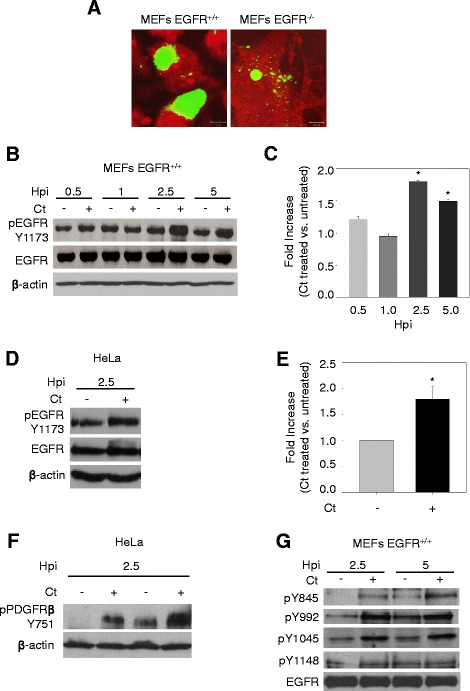
**EGFR is activated by** ***C. trachomatis*** **infection. (A)** MEFs EGFR^+/+^ and MEFs EGFR ^−/−^ cells were infected with *C. trachomatis* (*Ct)*. Note the small chlamydial inclusions (in apple green) formed in the MEFs EGFR^−/−^ cells in comparison to the inclusions formed in MEFs EGFR^+/+^ cells. Red color is the counterstaining of the host cell. **(B)**-**(F)** Phosphorylation of EGFR in *C. trachomatis*-infected cells. Monolayers of MEFs EGFR^+/+^ **(B)** and HeLa **(D)** with and without chlamydial infection were lysed at different hpi as indicated and immunoblotted with antibodies against pY1173-EGFR and EGFR antibodies. The immunoblots from three independent experiments were quantified for both MEFs **(C)** and HeLa cells **(E)** after normalization with β-actin used as loading control. A significant increase (P < 0.05) in phosphorylation of EGFR in MEFs EGFR^+/+^ **(C)** and HeLa cells **(E)** was observed at 2.5 hpi. **(F)** HeLa cells with and without chlamydial infection were lysed at 2.5 hpi. Two biological replicates were subjected to immunoblotting for pPDGFRβ (Y751) and β-actin as loading control. An increase in PDGFRβ phosphorylation was observed in *C. trachomatis*-infected cells compared with non-infected cells. **(G)** MEFs EGFR^+/+^ were infected with *C. trachomatis* for 2.5 h or 5 h. Western blotting was performed for comparing EGFR phosphorylation by *C. trachomatis* at various tyrosine residues. *C. trachomatis* induced phosphorylation was observed at all sites analyzed with the exception of Y1148 site.

**Figure 2:**
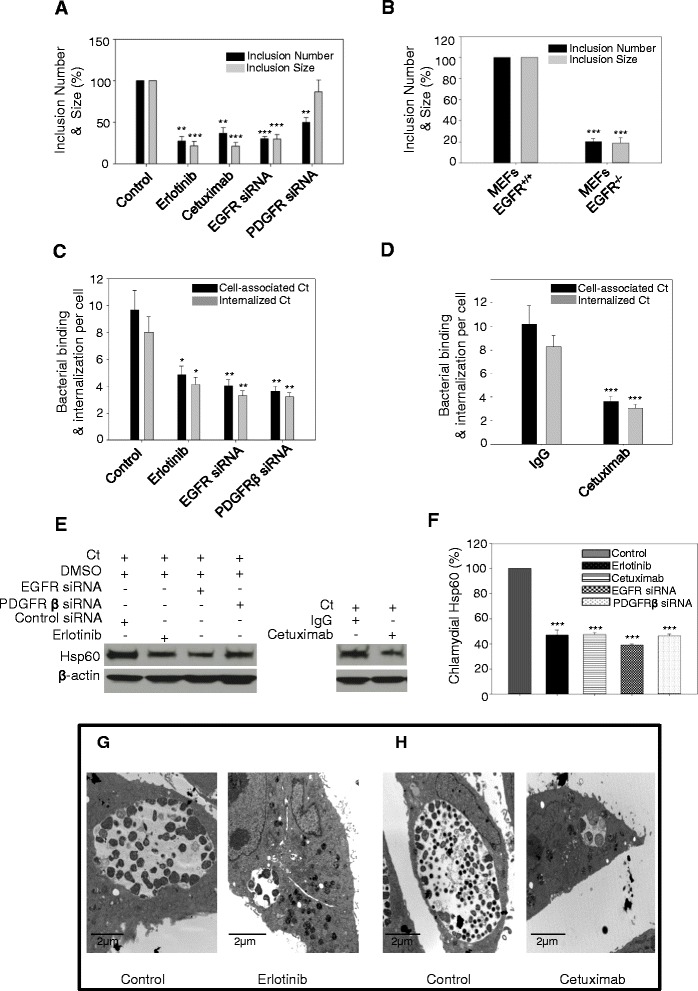
**EGFR is important for bacterial attachment and inclusion development. (A)** and **(B)** Effect of EGFR inhibition on number and size of inclusions. **(A)** HeLa cells treated with Erlotinib , Cetuximab, EGFR siRNA or PDGFRβ siRNA were infected with *C. trachomatis* for 24 h, fixed, analyzed by confocal microscopy and ImageJ software. Data from three independent experiments are expressed as percentage of total number of inclusions (black bar) or inclusion size (gray bar) in comparison to the control. **(B)** MEFs EGFR^+/+^ and MEFs EGFR^−/−^ were infected and processed as in **(A)**. Data are from three independent experiments. **(C)** and **(D)** Effect of EGFR inhibition on chlamydial attachment and entry into the host cell. HeLa cells treated with Erlotinib, EGFR siRNA and PDGFRβ siRNA **(C)** or Cetuximab **(D)**, were infected with *C. trachomatis* for 2.5 h and inside out staining was performed to differentially stain both external and internalized *C. trachomatis*. Data from three independent experiments are expressed as number of cell-associated bacteria (external + internalized *C. trachomatis*) and internalized *C. trachomatis* per infected host cell. **(E)** and **(F)** Levels of chlamydial Hsp60 antigen. HeLa cells treated with Erlotinib, Cetuximab, EGFR siRNA or PDGFRβ siRNA were infected with *C. trachomatis* for 2.5 h and Western blotting was performed with anti-chlamydial Hsp60 antibody. Quantification of the Western blots is from three independent experiments. **(F)**. β-actin was used as loading control. **(G)** and **(H)** Transmission electron micrographs of HeLa cells infected with *C. trachomatis*. HeLa cells treated with Erlotinib **(G)** and Cetuximab **(H)** with respective controls, were infected with *C. trachomatis* for 24 h and fixed for transmission electron microscopy. Note the typical large inclusions in the control chlamydia infected cells. In cells treated with Erlotinib and Cetuximab the inclusions are smaller and less mature. Scale Bar = 2 μm.

To assess the role of EGFR in *C. trachomatis* development, we initiated our studies by comparing the chlamydial inclusion formation between isogenic cell lines, MEFs EGFR^+/+^ (mouse embryonic fibroblasts) and EGFR null MEFs (MEFs EGFR^−/−^). Both cell lines were infected with chlamydial EBs and at 24 hours post infection (hpi) the cells were stained with chlamydial FITC-conjugated anti-lipopolysaccharide (LPS) mAb as described in the *Methods*. Confocal imaging was performed to visualize the development of chlamydial inclusions. Well-developed *C. trachomatis* inclusions were observed in MEFs EGFR^+/+^ while in the MEFs EGFR^−/−^ cells, the inclusions were significantly smaller in size in comparison to MEFs EGFR^+/+^ (Figure 1A, quantification is shown in Figure 2B). These initial results indicated a role of EGFR in *C. trachomatis* infection and prompted us to explore it further. We first examined whether *C. trachomatis* could induce EGFR phosphorylation in infected cells. To ensure the results were not biased by the selection of cell line, both HeLa cells and MEFs EGFR^+/+^ were used in these experiments. The MEFs EGFR^+/+^ cells were infected with chlamydial EBs and lysed at different time points ranging from 0.5 hpi to 5 hpi. We observed a significant 1.8-fold increase in phosphorylation of Y1173 in EGFR that peaked at 2.5 hpi (P < 0.05) (Figure 1B and C). Similar results were obtained in HeLa cells (Figure 1D and E), in which we also observed an increase in PDGFRβ phosphorylation (Figure 1F). We further analyzed the phosphorylation of EGFR at other tyrosine residues (Y845, Y992, Y1045 and Y1148). An increased phosphorylation was observed in *C. trachomatis*-infected MEFs EGFR^+/+^ (2.5 and 5 hpi) at all sites analyzed with the exception of Y1148 (Figure 1G). The results show that *C. trachomatis* can enhance EGFR activity and predicts a function of EGFR signaling in *C. trachomatis* infection.

**Figure 3:**
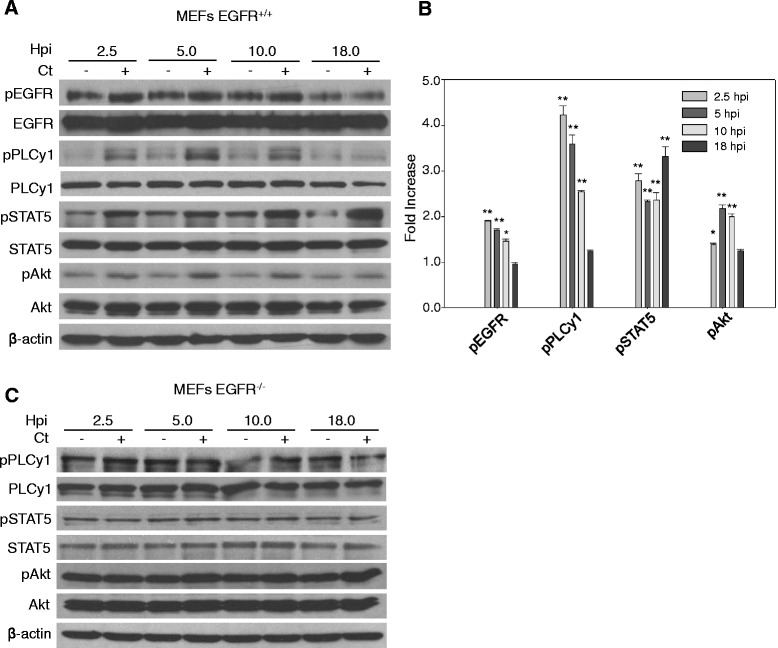
***C. trachomatis*** **activates EGFR downstream signaling. (A)** Monolayers of MEFs EGFR^+/+^ and **(C)** MEFs EGFR^−/−^ cells with and without chlamydial infection were lysed at 2.5, 5, 10 and 18 hpi. Cell lysates were immunoblotted with antibodies against phosphorylated and total EGFR, PLCγ1, Akt and STAT5. β-actin was used as loading control. Each phosphorylated protein was first normalized against the total protein and then the fold increase from –Ct to + Ct was calculated from three independent experiments **(B)**. An increased phosphorylation of EGFR, PLCγ1, Akt and STAT5 was observed in MEFs EGFR^+/+^ (P < 0.05) while no change was observed in the MEFs EGFR^−/−^ upon *C. trachomatis* infection.

**Figure 4:**
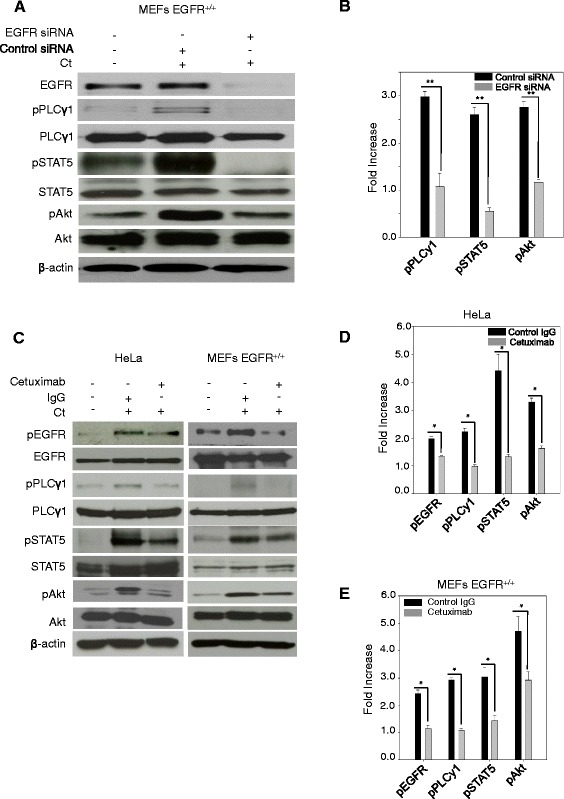
***C. trachomatis*** **-induced activation of PLCγ1, STAT5 and Akt is EGFR-dependent. (A)** and **(B)** MEFs EGFR^+/+^ were treated with either scrambled (control) or EGFR siRNA and then infected with *C. trachomatis*. Cells were lysed after 2.5 hpi and immunoblotted with antibodies against phosphorylated and total PLCγ1, STAT5 and Akt **(A)**. Western blots from three independent experiments were quantified. **(B)**. **(C-E)** HeLa and MEFs EGFR^+/+^ were treated with Cetuximab (an anti-EGFR antibody that blocks the binding of EGF to EGFR thus blocking receptor activation) followed by *C. trachomatis* infection. At 2.5 hpi the lysates were immunoblotted with antibodies against phosphorylated and total EGFR, PLCγ1, STAT5 and Akt **(C)**. Western blots from three independent experiments were quantified for both HeLa **(D)** and MEFs **(E)**. *C. trachomatis*-induced phosphorylation of PLCγ1, STAT5 and Akt was completely or partially abrogated in cells that were either depleted of EGFR (**A** and **B**; P < 0.01) or treated with Cetuximab (**C**-**E**; P < 0.05). β-actin was used as loading control.

EGFR activation in response to extracellular cues (e.g., EGF ligand) is known to activate PI3K/Akt, PLCγ1 (phospholipase Cγ1) and STAT proteins (signal transducers and activators of transcription) [36-38]. To determine whether *C. trachomatis*-induced EGFR phosphorylation can also activate its downstream effector proteins, the phosphorylation of PLCγ1 (Y783), Akt (S473) and STAT5 (Y694) was monitored in MEFs EGFR^+/+^ and EGFR^−/−^ cells infected with chlamydial EBs at time points ranging from 2.5 hpi to 18 hpi. As shown in Figure 3A and the quantification in Figure 3B, *C. trachomatis* infection induced a significant increase in phosphorylation of EGFR and its downstream targets, PLCγ1, Akt and STAT5 at 2.5, 5 and 10 hpi (P < 0.05). At 18 hpi, the phosphorylation of EGFR, Akt and PLCγ1 returned close to the basal level. Interestingly, the phosphorylation of STAT5 persisted at 18 hpi, presumably due to delayed kinetics or secondary activation subsequent to primary stimulus, an aspect that will be investigated in future studies. The EGFR dependence of these phosphorylation events was further confirmed by the experiments in MEFs EGFR^−/−^ cells. Under these conditions, *C. trachomatis* infection at the same time points did not induce an increase in phosphorylation of PLCγ1, Akt and STAT5 proteins (Figure 3C). Combined with our previous observation of phenotypically smaller inclusions formed in MEFs EGFR^−/−^ cells (Figure 1A), these findings led us to hypothesize that activation of an EGFR-dependent signaling axis was essential for establishing a successful *C. trachomatis* infection. To further confirm that the activation of these proteins was *C. trachomatis*- and EGFR-dependent, we investigated the *C. trachomatis*-induced activation of PLCγ1, STAT5 and Akt in MEF EGFR^+/+^ and HeLa cells using multiple methods of EGFR inhibition. MEFs EGFR^+/+^ cells were treated with EGFR siRNA for 48 h, then infected with *C. trachomatis* for 2.5 h and tested for activation of PLCγ1, STAT5 and Akt. Chlamydial infection resulted in increased phosphorylation of all three proteins in cells treated with control siRNA but not in cells treated with EGFR siRNA (Figure 4A and B; P < 0.01). *C. trachomatis*-induced phosphorylation of EGFR, PLCγ1, STAT5 and Akt was also inhibited by treatment of HeLa and MEFs EGFR^+/+^ with Cetuximab, a monoclonal antibody that binds to the extracellular domain of EGFR and specifically blocks EGFR functions (Figure 4C-E). These studies confirmed EGFR activation as one of the upstream regulatory events in the phosphorylation of PLCγ1, STAT5 and Akt signaling in *C. trachomatis*-infected cells.

### EGFR is essential for the formation of mature chlamydial inclusions

Next, we assessed the contribution of EGFR to the formation of *C. trachomatis* inclusions in HeLa cells and MEFs. EGFR was inhibited by using Erlotinib, Cetuximab or EGFR siRNA. Erlotinib is a small molecule inhibitor that targets the intracellular kinase domain of EGFR, while Cetuximab blocks the binding of EGF to its cognate receptor and thus blocks receptor activation [39,40]. Effective inhibition of EGFR phosphorylation by Erlotinib or Cetuximab was confirmed by Western blot (Additional file 1: Figure S1, and S2, and Figure 4C). Similarly, Western blot analysis was performed to confirm the depletion of EGFR protein in HeLa and MEFs treated with EGFR siRNA (Additional file 1: Figure S3 and Figure 4A). HeLa cells with or without EGFR inhibition (protein depletion or inhibition of function), were infected with *C. trachomatis*. The cells were immunostained using anti-chlamydial LPS mAb at 24 hpi and analyzed by confocal imaging to quantify the size and number of inclusions. In comparison to control (DMSO, IgG or control siRNA treated cells), there was a significant decrease in both the number and size of chlamydial inclusions under all treatment conditions (P < 0.01 to P < 0.001; Figure 2A). Use of multiple approaches to inhibit EGFR discounts the possibility of observing these results because of an unspecific interaction of the inhibitors with non-target molecules, a potential caveat of using pharmacological agents [41]. The decreased chlamydial infection upon EGFR inhibition was further confirmed by monitoring the chlamydial Hsp60 using Western blot analysis (Additional file 1: Figure S4). To ensure that Erlotinib and Cetuximab treatments did not affect the viability of chlamydial EBs, we infected HeLa cells with EBs that were pretreated with Erlotinib or Cetuximab. At 24 hpi the cells were lysed and blotted using antibodies against chlamydial Hsp60. The Hsp60 antigen load in the cells infected with drug-treated EBs was comparable to the infection by the untreated EBs (Additional file 1: Figure S5), confirming that EGFR inhibitors did not affect the viability of EBs in these experiments. Additional studies were performed to ensure that the poor inclusion development was not due to loss of the host cells’ viability during Erlotinib treatment. The highest concentration (25 μM) and maximum duration (24 h) of Erlotinib treatment did not reduce the viability of HeLa cells (Additional file 1: Figure S6).

Because we observed PDGFRβ phosphorylation triggered by *C. trachomatis* infection (Figure 1F) and the role of PDGFRβ has been established in *C. muridarum* attachment to host cells [19], we also investigated the formation of chlamydial inclusions in cells treated with PDGFRβ siRNA. The depletion of PDGFRβ in HeLa cells treated with PDGFRβ siRNA was confirmed by Western blot (Additional file 1: Figure S7). Similar to EGFR, depletion of PDGFRβ decreased the number of inclusions in host cells (P < 0.01); however, unlike EGFR, the size of the inclusions was not significantly affected by PDGFRβ siRNA treatment (Figure 2A and Additional file 2: Figure S13). These results were further substantiated by experiments in MEFs EGFR^−/−^ cells, which showed similar results to the EGFR-inhibited HeLa cells (Figure 2B and Additional file 1: Figure S8). We then examined the possible role of EGFR in the bacterial attachment to the cell surface and its subsequent internalization, during the early stage of *C. trachomatis* infection. The PDGFRβ siRNA treated HeLa cells were included as control in these experiments. HeLa cells with or without EGFR and PDGFRβ inhibition were infected with *C. trachomatis* for 2.5 h and processed for inside-out staining to differentially quantify external and internalized bacteria. At 2.5 hpi a significant decrease in cell-associated bacteria (external and internal) was observed (P < 0.05 to P < 0.001) in both EGFR and PDGFRβ inhibited cells (Figure 2C and D). Since more than 80% of the cell-associated bacteria were successfully internalized into the host cell (Figure 2C and D), these results point to defects in bacterial attachment to the host cell as a main cause for the decrease in overall chlamydial internalization. The results were further confirmed by Western blot analysis of chlamydial Hsp60 (P < 0.001; Figure 2E and F).

The small inclusions formed upon inhibition of EGFR were examined in detail by transmission electron microscopy experiments. Large inclusions were observed in HeLa cells infected with *C. trachomatis* whereas the Erlotinib and Cetuximab treated HeLa cells contained small inclusions (Figure 2G and H).

**Figure 5:**
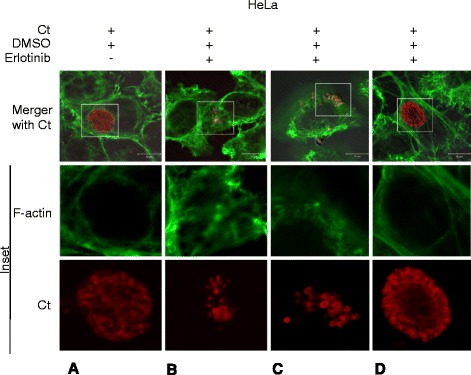
**EGFR is essential for development of chlamydial inclusion post-bacterial entry. (A-D)** HeLa cells were infected with *C. trachomatis* for 24 h. In panels B-D, EGFR inhibitor Erlotinib was added at 2.5, 5 and 18 hpi, respectively. Under all conditions the total time for *C. trachomatis* infection was 24 h followed by fixing, and processing for confocal microscopy. F-actin was detected with Alexa Fluor 488-phalloidin (green) and chlamydial inclusions were detected using anti-chlamydial LPS antibody (red). Well-developed *C. trachomatis* inclusions were observed in the DMSO treated cells **(A)** and in cells treated with Erlotinib at 18 hpi **(D)**; while incomplete inclusion development and bacterial aggregates were observed in cells treated with Erlotinib at 2.5 hpi **(B)** and 5 hpi **(C)**. F-actin staining of HeLa cells with Erlotinib treatment but without *C. trachomatis* infection is shown in Additional file 2: Figure S12. Scale bar - 10 μm.

To further distinguish between the role of EGFR in bacterial attachment from the growth-associated consequences during *C. trachomatis* infection, experiments were performed in which EGFR inhibitor (Erlotinib) was added at different time points (2.5, 5 and 18 hpi), post bacterial infection. Under all conditions the total time of infection with *C. trachomatis* was 24 h. EGFR inhibition after 2.5 and 5 hpi, impaired regular inclusion formation and resulted in formation of numerous bacterial aggregates (Figure 5B and C, respectively). On the other hand, EGFR inhibition at 18 hpi did not significantly affect the inclusion development (Figure 5D). These observations correlate well with the pattern of EGFR signaling shown in Figure 3A and B. The results were further confirmed by Western blot analysis of chlamydial Hsp60. Significantly lower expression of Hsp60 was noted in cells treated with Erlotinib at 2.5 hpi compared with Erlotinib addition at 18 hpi (cells harvested at 24 hpi, Additional file 1: Figure S9). Together, the results described above show that EGFR has discrete functions both at the level of bacterial attachment/internalization and subsequent inclusion development.

### EGFR regulates intracellular calcium during *C. trachomatis* infection

**Figure 6:**
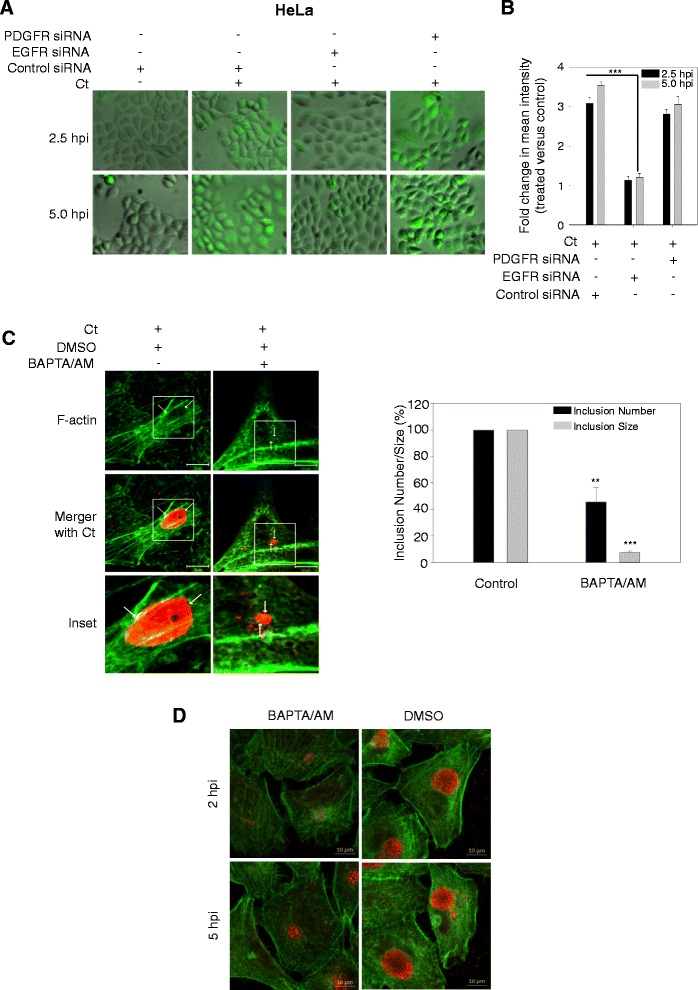
**EGFR is required for** ***C. trachomatis*** **-induced calcium mobilization. (A)** and **(B)** Calcium mobilization induced by *C. trachomatis* infection. HeLa cells treated with control siRNA, EGFR siRNA or PDGFRβ siRNA were infected with *C. trachomatis* for 2.5 h or 5 h and stained with Fluo-4 AM for visualization of calcium (Ca^2+^) by fluorescence microscopy. The fluorescence intensity of calcium staining from three independent experiments was quantified using ImageJ **(B)**. **(C)** Inclusion development and organization of F-actin at the chlamydial inclusion periphery. HeLa cells were pre-treated with BAPTA/AM (a calcium chelator) for 1h followed by infection with *C. trachomatis* for 24 h, fixed, and processed for confocal microscopy. Data from three independent experiments were analyzed for number and size of inclusions that were significantly reduced in BAPTA/AM treated cells (left panel). F-actin was detected with Alexa Fluor 488-phalloidin (green) and chlamydial inclusions were detected using anti-chlamydial LPS antibody (red) (right panel). Note the diffused assembly of F-actin at the inclusion periphery (arrow). **(D)** Inclusion development in post infection BAPTA/AM treated cells. HeLa cells were infected with *C. trachomatis* for 24 h. BAPTA/AM or control DMSO was added at 2 or 5 hpi. Under all conditions the total time for *C. trachomatis* infection was 24 h followed by fixing, and processing for confocal microscopy. F-actin was detected with Alexa Fluor 488-phalloidin (green) and chlamydial inclusions were detected using anti-*C. trachomatis* EB antibody (red). Data is representative of two independent experiments. Note the impaired inclusion development in BAPTA/AM treated cells in comparison to the DMSO treated cells. F-actin staining for HeLa cells with BAPTA/AM treatment but without *C. trachomatis* infection is also shown in Additional file 2: Figure S12. Scale bar - 10 μm.

The electron micrographs of the inclusions formed in EGFR-inhibited cells (Figure 2G & H) were reminiscent of previously reported type of inclusions formed upon inhibition of calcium binding protein calmodulin [42]. Since EGFR is known to regulate cellular calcium response and calcium signaling is pivotal for numerous bacterial infections [43,44], we next tested whether EGFR could be involved in *C. trachomatis*-induced calcium release in host cells. HeLa cells were treated with control siRNA, EGFR siRNA or PDGFRβ siRNA followed by *C. trachomatis* infection. At 2.5 and 5 hpi cells were analyzed by fluorescence microscopy for intracellular calcium. At 2.5 and 5 hpi, a significant increase in calcium was observed upon *C. trachomatis* infection in control siRNA and PDGFRβ siRNA treated cells but not in EGFR siRNA treated cells (Figure 6A and B; P < 0.001). In another set of experiments, Erlotinib was added at 2.5 and 5 hpi and cells were incubated for up to 24 hpi when they were stained for calcium. A significant drop in calcium signal was observed in *C. trachomatis*-infected cells that had been treated with Erlotinib at 2.5 and 5 hpi (Additional file 2: Figure S10). Examination of these results in combination with the observations described in Figure 5, suggest that EGFR-induced calcium release is necessary for the development of *C. trachomatis* inclusions. Next, we mimicked a calcium deficient environment by treating HeLa cells with the calcium chelator BAPTA/AM for 1 h followed by *C. trachomatis* infection for 24 h. A significant decrease in both inclusion size and number was noted, similar to the conditions of EGFR inhibition in HeLa cells (Figure 6C). Impaired inclusion formation was also observed in *C. trachomatis*-infected cells treated with BAPTA/AM at 2 and 5 hpi (more severe in 2 hpi BAPTA/AM treated cells) compared with the control DMSO treated cells (Figure 6D). Intriguingly, the addition of Ionomycin (a calcium ionophore) to EGFR siRNA treated cells was not able to rescue the formation of chlamydial inclusion (Additional file 2: Figure S11). This shows that a coordinated and synchronized regulation of EGFR-dependent calcium release along with other factors regulated by EGFR are required for formation of inclusions within the host cells. We hypothesized that one of these factors could be F-actin; the reasoning and experiments to address this hypothesis are described next.

### EGFR is essential for F-actin assembly around chlamydial inclusions

**Figure 7:**
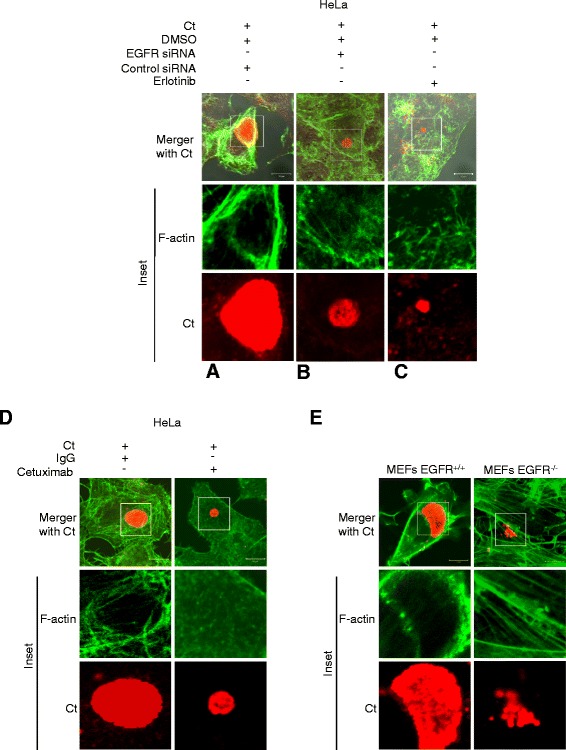
**EGFR is required for the reorganization of F-actin at the periphery of chlamydial inclusions. (A-C)** Organization of F-actin at the chlamydial inclusion periphery. HeLa cells treated with **(A)** control siRNA, **(B)** EGFR siRNA or **(C)** Erlotinib, were infected with *C. trachomatis* for 24 h, fixed, and processed for confocal microscopy. F-actin was detected with Alexa Fluor 488-phalloidin (green) and chlamydial inclusions were detected using anti-chlamydial LPS antibody (red). Note the distinct assembly of F-actin at the inclusion periphery in **(A)** which is altered upon inhibition of EGFR **(B** and **C)**. **(D)** HeLa cells treated with IgG or Cetuximab were infected with Ct for 24 h, fixed, and processed for confocal microscopy. F-actin was detected with Alexa Fluor 488-phalloidin (green) and chlamydial inclusions were detected using anti-chlamydial LPS antibody (red). Note the distinct assembly of F-actin at the inclusion periphery in IgG treated cells and the lack of it in Cetuximab treated cells. Scale bar - 10 μm. **(E)** Organization of F-actin at the chlamydial inclusion periphery in MEFs EGFR^+/+^ and MEFs EGFR^−/−^. MEFs EGFR^+/+^ and MEFs EGFR^−/−^ were infected and processed as in **(A)**. F-actin is noticeably rearranged at the chlamydial inclusion periphery in the MEFs EGFR^+/+^ but not in the MEFs EGFR^−/−^ cells.

Cytoskeletal elements like F-actin and intermediate filaments have been shown to form a dynamically regulated scaffold around the chlamydial inclusions [18]. Interestingly, a unique feature of EGFR that is not present in other receptor tyrosine kinases (e.g., PDGFR or FGFR) is the presence of an F-actin binding domain involved in the internalization of activated EGFR [45]. Independent studies have implicated EGF-induced PLCγ1 activation in both calcium mobilization and cytoskeleton remodeling [31,43,46]. Our results demonstrate EGFR-dependent PLCγ1 activation and calcium mobilization upon *C. trachomatis* infection (Figures 4 and 6). To examine the role of EGFR in arrangement of F-actin at the inclusion periphery, HeLa cells treated with Erlotinib, Cetuximab, and EGFR siRNA were infected with *C. trachomatis* for 24 h and processed for confocal microscopy to visualize the intracellular arrangement of F-actin. We observed formation of a distinct F-actin ring at the inclusion periphery in *C. trachomatis* infected control cells (DMSO, control siRNA or IgG control) (Figure 7A and D). In the EGFR siRNA treated cells, the arrangement of F-actin around the *C. trachomatis* inclusion was either diffused or disorganized (Figure 7B). Similar results were observed in the Erlotinib and Cetuximab treated HeLa cells (Figure 7C and D), as well as in the MEFs EGFR^−/−^ cells (Figure 7E).

**Figure 8:**
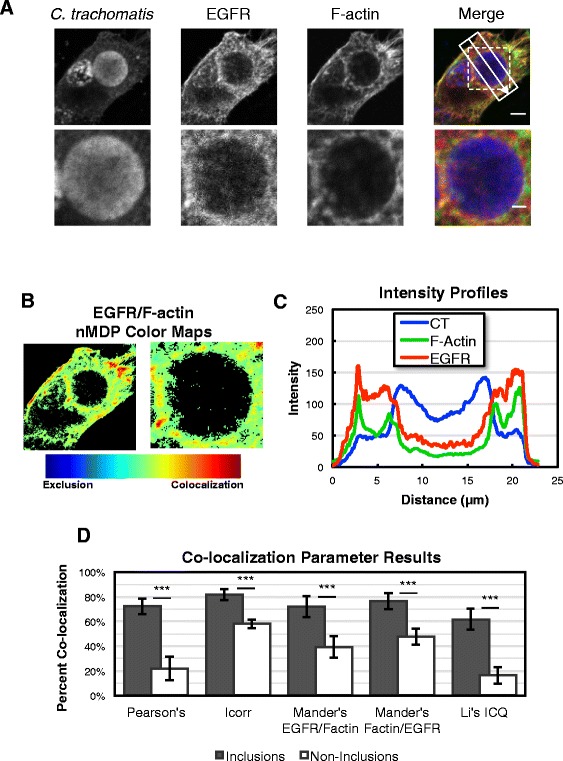
**Co-localization of EGFR and F-actin at the periphery of** ***C. trachomatis*** **inclusion. (A)** HeLa cells were infected with *C. trachomatis* for 24 h, fixed and processed for confocal imaging to detect *C. trachomatis* (blue), F-actin (green) and EGFR (red). The merged image shows co-localization of EGFR and F-actin as illustrated by the yellow signal. Dashed box represents the inclusion area, solid box represents area and direction of intensity profile measurement in **(C)**. Scale bars are 5 μm and 2 μm in whole cell and inclusion area images, respectively. **(B)** nMDP color maps showing heat maps of co-localization areas in whole cell and inclusion area images. Both cell and inclusion boundaries show similar evidence of co-localization ranging from moderate to intense. **(C)** Intensity profiles of *C. trachomatis*, EGFR, and F-actin from cell boundary to cell boundary across the inclusion. EGFR and F-actin signals rise and fall in similar patterns along the inclusion boundary (located at approximately 6 μm and 18 μm on the x-axis) indicating co-localization in a similar manner as at the cell boundary (located at approximately 3 μm and 21 μm on the x-axis). **(D)** Comparison of co-localization parameters between inclusion area images and non-inclusion area images (details in the legend for Additional file 3: Figures S14-S20). All five parameters show significant increase of co-localization in inclusion areas compared with non-inclusion areas. Data presented represent the mean ± standard deviation; n = 8 images within each subset, ***p < 0.001.

Since EGFR is an F-actin binding protein, we performed additional experiments to investigate whether EGFR co-localizes with F-actin ring at the periphery of inclusion. HeLa cells were infected with *C. trachomatis* and at 24 hpi the cells were stained for *C. trachomatis* EB, EGFR and F-actin (Figure 8A). Co-localization of EGFR and F-actin at the periphery of *C. trachomatis* inclusion was evidenced by overlapping fluorescence signals (Figure 8A, Merge). The normalized mean deviation product (nMDP) [47] was calculated for each pixel in the image to identify regions of intense co-localization or exclusion within the image. The resulting nMDP color maps of the whole cell and the inclusion area (dashed box in Figure 8A) show areas with co-localization ranging from moderate to intense, with no areas of exclusion (Figure 8B). The intensity profile for a cross-section in Figure 8A, which includes from top left to bottom right (left to right in the intensity profile plot) shows clear enrichment of EGFR and F-actin at the periphery of inclusion as well as the cell membrane (Figure 8C). Quantitative processing of the image data shown in Figure 8 was performed using a number of independent algorithms [48], which are described in the legend for Additional file 3: Figures S14-S20. Similar analysis was applied to other 7 inclusion areas and 8 non-inclusion areas (cells that were not infected with *C. trachomatis*) and all five parameters show significant increase of EGFR and F-actin co-localization in inclusion areas compared with non-inclusion areas (p < 0.001, Figure 8D). Additional representative images and analyses are shown in Additional file 3: Figures S15-20. Collectively, all quantitative analyses show strong and statistically significant evidence of co-localization of EGFR and F-actin at the periphery of inclusion.

## Discussion

As an intracellular pathogen, *C. trachomatis* has developed an arsenal of molecular tools that enables it to hijack signaling and metabolic pathways of the host cell and establish an intracellular niche favorable to its development. An extensive network of interactions exists between *C. trachomatis* and host proteins to facilitate bacterial attachment and entry and *C. trachomatis* development in the host cell. *C. trachomatis* can interact with and modulate the activity of numerous cell surface receptors to promote attachment and entry into the host cell. EGFR is an important cell surface receptor tyrosine kinase with a central role in cell growth, proliferation and migration [49]. The function of EGFR was studied in a number of bacterial and viral infections (e.g., *C. pneumoniae, Pseudomonas aeruginosa*, *Neisseria gonorrhoeae,* HPV); however, it has not been thoroughly investigated in relation to *C. trachomatis* pathogenesis. We provide here the first evidence that *C. trachomatis* has the ability to upregulate EGFR activity in host cells and establish EGFR as a critical effector molecule in the formation of chlamydial inclusions within the host cells.

We demonstrate that *C. trachomatis* induces an increase in EGFR phosphorylation and that inhibition of EGFR phosphorylation or depletion of EGFR protein impairs *C. trachomatis* attachment and its development in the host cells. In 2008, Elwell *et al.* analyzed both PDGFRβ and EGFR phosphorylation in HeLa cells infected with *C. muridarum* [19]. While there was an increased phosphorylation of PDGFRβ, a change in phosphorylation of EGFR was not observed in this study. The discrepancy in results could be due to differences in the time points (hpi) investigated, MOI and *Chlamydia* species. Our results are further supported by the EGFR-dependent increase in phosphorylation of downstream targets like Akt, STAT5 and PLCγ1 in *C. trachomatis* infected cells. Phosphorylation of Akt during chlamydial infection is well documented [27-29] and previous studies reported that activation of the PI3K/Akt pathway blocks the cytochrome c release from the mitochondria and delays apoptosis thereby promoting chlamydial growth and survival inside the host cell [50]. The lack of Akt phosphorylation in EGFR^−/−^ cells demonstrates EGFR as the upstream regulator of Akt phosphorylation, which was not known before. With respect to STAT5 activation, a recent report showed that the interaction of EGFR with STAT5 in the nucleus could lead to activation of Aurora-A gene expression [37], and subsequent chromosomal instability. Interestingly, *C. trachomatis* infection was previously linked to centrosome defects [51,52]. Thus, a mechanistic connection could exist between *C. trachomatis*-induced activation of EGFR and STAT5 and the centrosome abnormalities observed in *C. trachomatis* infections; this remains to be investigated.

In this study, we have followed-up on EGFR-dependent activation of PLCγ1. It is well known that EGF binding to EGFR results in activation of PLCγ1. The subsequent production of IP_3_ is linked to activation of IP3 receptor (IP3R) and intracellular calcium mobilization [53]. There is extensive evidence that links EGFR activation to this process [43,46] and we demonstrate here that inhibition of EGFR abrogates the *C. trachomatis*-induced increase in intracellular calcium flux. Also, removal of calcium by chelation resulted in marked reduction in the size and number of chlamydial inclusion similar to EGFR inhibition. This is consistent with previous studies, which have reported localization of intracellular calcium stores and regulators of calcium release and uptake (IP3R and SERCA2, respectively) in the vicinity of *C. trachomatis* inclusions and formation of immature inclusions upon inhibition of calcium binding proteins like calmodulin [42,54]. However, the failure to restore *C. trachomatis* inclusion development by addition of a calcium ionophore (e.g., Ionomycin) indicated that other EGFR-regulated processes might contribute to *C. trachomatis* inclusion development in conjunction with calcium.

*C. trachomatis* is known to utilize cytoskeletal elements like F-actin and intermediate filaments to form a dynamically regulated scaffold that maintains the structural integrity of the chlamydial inclusions [18]. A unique feature of EGFR that distinguishes it from PDGFRβ is its ability to regulate actin polymerization through its F-actin binding domain. EGFR and F-actin interaction was previously reported to play an active role in EGFR internalization and has been proposed to localize EGFR signaling at specific loci within the cell [45,55]. Our data show distinct co-localization of EGFR with the F-actin ring around the chlamydial inclusion and interruption in the formation of F-actin rings around the chlamydial inclusions in both calcium depleted and EGFR inhibited cells. These results are consistent with the function of PLCγ1 and calcium mobilization in cytoskeletal remodeling [31,32]. Rho family of small GTPases has also been reported to participate in the EGFR-dependent regulation of cytoskeletal remodeling [55] and previous findings have shown recruitment of these molecules at the inclusion periphery [18] Therefore, EGFR interaction with F-actin may function to nucleate the assembly of signaling protein complexes for cytoskeletal remodeling required for inclusion development.

EGFR-dependent regulation of F-actin and calcium release can affect inclusion formation at multiple stages of *C. trachomatis* infection. First, it can have a direct effect on the bacterial attachment and entry. Consistent with this, our data indicate that EGFR inhibition significantly impairs the bacterial attachment to the host cell surface at a level comparable with inhibition of PDGFRβ. Recently, Molleken *et al.* demonstrated that EGFR inhibition (by another EGFR tyrosine kinase inhibitor AG1478) reduced *C. pneumoniae* EB internalization to HEp-2 cells but did not affect EB attachment [25]. The discrepancy could be due to differences in the *Chlamydia* species, host cell lines and the dose and type of EGFR inhibitors used in these studies. Nevertheless, the defect in bacterial attachment seen in our studies could be due to the inability to bind to EGFR and/or the defective EGFR signaling. Since Erlotinib, as an EGFR tyrosine kinase inhibitor, does not directly compete with *C. trachomatis* for binding to EGFR, our results suggest that *C. trachomatis* attachment is not only mediated by its binding to EGFR, but also facilitated by the downstream signaling events. Early events of *C. trachomatis* infection (1–2 hpi) involve accumulation of tyrosine phosphorylated proteins at the site of entry [16,17,56,57], cytoskeletal remodeling and reorganization of microvilli at the cellular surface for *C. trachomatis* uptake [58,59]. We propose that *C. trachomatis*-activated EGFR can trigger changes in cytoskeletal rearrangement and microvilli extensions that favor *C. trachomatis* attachment and entry. Whether EGFR activation is the direct result of EBs binding to the extracellular domain of EGFR or occurs indirectly (e.g., through activation of Src tyrosine kinase), it remains to be investigated in future studies. Alternatively, reduced *C. trachomatis* attachment to Erlotinib-treated host cells could be due to changes in EGFR subcellular localization or EGFR affinity to its ligand. However, in our imaging studies, we did not detect any significant changes in EGFR cell surface localization in the presence of Erlotinib, consistent with another report [60]. Although other EGFR tyrosine kinase inhibitors (e.g. Gefitinib, AG1478) have been shown to alter EGFR interaction with its ligand [61,62], whether Erlotinib could change the affinity of EGFR to its ligand remains to be investigated. However, the Molleken *et al.* studies, which used AG1478 would suggest that this mechanism is unlikely and that the attachment of EBs to host cell is impaired by inhibition of EGFR downstream signaling. It should be noted that entry of *C. trachomatis* in the host cell could use multiple routes. Our data supports this statement since independent inhibitions of either EGFR or PDGFRβ affect the bacterial attachment and neither of the two receptors can compensate for the other. The second stage at which EGFR can control the inclusion development is at the local aggregation of *C. trachomatis* inside the cell. In fact, experiments in which EGFR was inhibited post chlamydial invasion, showed decreased intracellular calcium (Additional file 2: Figure S10) as well as numerous bacterial aggregates (Figure 5), perhaps stalled at the stage of fusion of bacterial inclusions. As discussed above, a number of studies have indicated a role of calcium in aggregation of *C. trachomatis* and F-actin rearrangement [32,54]. The EGFR-dependent regulation of calcium and F-actin not only provides the dynamic structural scaffold for the expanding inclusion but can also aid in the nutrient uptake required for replicating bacteria since the F-actin and microtubules are known to be essential for vesicular trafficking and intracellular motility [63]. Interference with the F-actin arrangements by inhibiting EGFR can therefore impair the maturation of chlamydial inclusions.

## Conclusions

**Figure 9:**
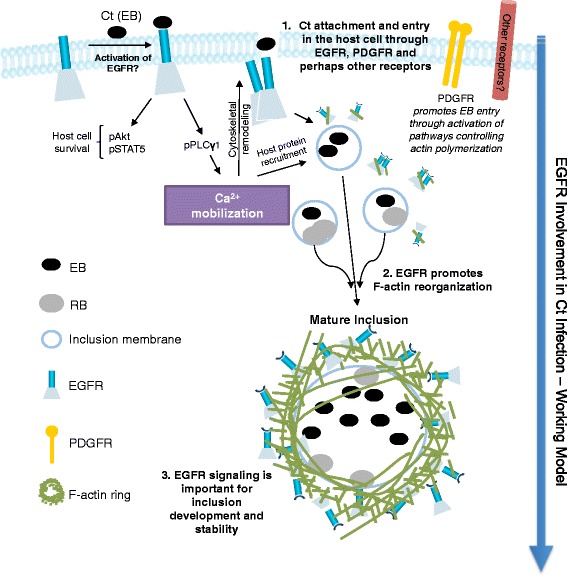
**Proposed model of EGFR involvement at various stages of** ***C. trachomatis*** **infection.** *C. trachomatis* infection induces EGFR phosphorylation and activation of downstream targets like Akt, STAT5 and PLCγ1. The direct activation of EGFR by EBs binding to the extracellular domain of EGFR is shown here but other indirect mechanisms of EGFR activation are possible as well. Activation of EGFR signaling can upregulate host cell survival mechanisms and induce increased calcium mobilization. EGFR co-localization with F-actin is suggestive of a possible direct role of EGFR in formation of F-actin rings at the periphery of inclusion. The increased calcium signaling can have several functions ranging from cytoskeletal remodeling to recruitment of host proteins at the inclusion membrane. The EGFR-induced formation of F-actin ring and other cytoskeletal elements at the inclusion periphery can further aid in inclusion expansion via vesicular trafficking and nutrient uptake.

In summary, the studies included here show that chlamydial infection upregulates EGFR activity in host cells. This results in activation of downstream effectors of EGFR such as PLCγ1, Akt and STAT5. We demonstrate that EGFR and EGFR-mediated signaling play a critical role in both *C. trachomatis* attachment and development of *C. trachomatis* inclusions in host cells through mechanisms that involve EGFR-dependent regulation of calcium release, actin cytoskeleton rearrangement, and EGFR co-localization with F-actin at the inclusion periphery. These findings shed light on the complexity of *C. trachomatis*-host cell interactions, which when solved may open new venues to treat *C. trachomatis* infections and *C. trachomatis*-associated diseases. These results form the basis of the model we propose in Figure 9, which will be further evaluated in the future. In this model, EGFR plays a key role in the early and middle stages of *C. trachomatis* infection wherein the EGFR mediated calcium signaling and F-actin remodeling are central to the establishment of a successful *C. trachomatis* infection inside the eukaryotic cells. The model shows the direct activation of EGFR by EBs binding, but as discussed above other mechanisms are possible and warrant investigation.

## Methods

### Reagents

Antibodies were obtained from the following sources: goat anti-chlamydial LPS, goat anti - *C. trachomatis* EB (Meridian Life Sciences, Saco, ME, USA); FITC conjugated anti-chlamydial EBs (Fitzgerald, MA, USA), rabbit anti-EGFR, rabbit anti-PLCγ1, rabbit anti-pPLCγ1, rabbit anti-Stat5, rabbit anti-pStat5, rabbit anti-Akt, rabbit anti-pAkt, rabbit anti-pPDGFRβ (Y751), rabbit anti-EGFR (Alexa Fluor 594 conjugate) and rabbit anti-β actin (Cell Signaling, Danvers, MA, USA); rabbit anti-pEGFR (Y1173), rabbit anti-PDGFRβ, mouse anti-chlamydial Hsp60 and rabbit anti-mouse IgG HRP antibodies (Santa Cruz Biotechnology, Santa Cruz, CA, USA); rabbit anti-pEGFR antibodies (Y845, Y992, Y1045, Y1148; Millipore, Temecula, CA, USA); donkey anti-goat IgG H&L (Alexa Fluor 405) (Abcam, Cambridge, MA, USA); anti-EGFR-Alexa Fluor 488 antibody (Millipore, Temecula, CA, USA), goat anti-rabbit IgG HRP, FITC-conjugated anti-rabbit antibody and rhodamine red conjugated anti-goat secondary antibodies (Jackson Laboratories, West Grove, PA, USA). EGFR siRNA (human and mouse), PDGFRβ siRNA (human), control siRNA and siRNA transfection reagents were obtained from Santa Cruz Biotechnology, Santa Cruz, CA, USA and Dharmacon USA. DMEM, DMEM (Ca^++^ free), FCS, FBS and Alexa Fluor 488 phalloidin and Fluo-4 AM were purchased from Invitrogen, Grand Island, NY, USA. Pathfinder Chlamydia Culture Confirmation System was purchased from BioRad, Hercules, CA, USA. HBSS and PBS were purchased from Lonza, Walkersville, MD, USA. DEAE dextran, BAPTA/AM, Ionomycin, and cyclohexamide were purchased from Sigma Aldrich, St. Louis, MO, USA. Cell Proliferation Kit I (MTT) and Fast Start Universal SYBR green (Rox) were obtained from Roche, Indianapolis, IN, USA. Erlotinib was purchased from Selleck Chemicals LLC, Houston, TX, USA and Cetuximab was a kind gift from Dr. Katerina Politi (Department of Pathology, Yale University). Bicinchoninic acid (BCA) assay for protein quantification was purchased from Thermo Scientific, Rockford, IL, USA. ECL Plus Western blotting detection reagent was purchased from Perkin Elmer, Waltham, MA, USA.

### Cell culture

*Chlamydia trachomatis* strain D, HeLa and NIH 3T3 were purchased from ATCC. Mouse embryonic fibroblasts (MEFs EGFR^+/+^ and EGFR^−/−^) were kindly provided by Dr. Jennifer Grandis (University of Pittsburg) [64]. HeLa and MEFs were cultured using DMEM + 10% FBS. NIH 3T3 cells were cultured in DMEM + 10% FCS. All cell lines were maintained at 37°C and 5% CO_2_.

### Propagation of *Chlamydia* and infections

*Chlamydia trachomatis* strain D (*C. trachomatis*) was propagated in HeLa cells grown in complete DMEM containing cyclohexamide (2 μg/ml). After 48 h, infected cells were harvested in sucrose-phosphate-glutamate (SPG) buffer, ruptured by vortexing with 3mm glass beads. EBs were purified using previously described methods [28]. The resulting bacterial pellet was resuspended in cold SPG buffer with a 21 to 22-gauge injection needle and stored in aliquots at −80°C. For infection, chlamydial EBs were added to cells in monolayer (80% confluence) at a multiplicity of infection (MOI) of 2–10 for all studies included here. Centrifugation was not used during the infection.

### Immunoblotting

Cells were harvested and lysed in modified RIPA buffer (50 mM Tris, 150 mM NaCl, 1% sodium deoxycholate, 1% NP-40, 1 mM sodium fluoride) supplemented with protease inhibitor cocktail and phosphatase inhibitor tablet (Roche). For Western blotting of *C. trachomatis* antigens, the *C. trachomatis*-infected cells were lysed in 20 mM HEPES buffer (pH 8.0) containing 8 M urea supplemented with protease inhibitors. Cell lysates were incubated on ice for 1 h and then sonicated briefly. The soluble protein fraction was collected by centrifugation at 10,000 rpm. Total protein was estimated using the BCA method and equal amounts of proteins (10–20 μg) were processed for immunoblotting. Proteins were resolved on 10% SDS polyacrylamide gel and transferred onto a nitrocellulose membrane. The blot was blocked using 3% BSA and incubated with the indicated antibodies. ECL was used to detect the proteins according to the manufacturer’s instructions.

### siRNA transfections

Cells were grown to 60% confluency followed by transfection with EGFR siRNA/PDGFRβ siRNA or control siRNA (Santa Cruz/Dharmacon) according to manufacturer's protocol. After 24 h, transfected cells were replated for a second round of transfection. After another 24 h, cells were infected with *C. trachomatis* and incubated for different time intervals (siRNA was maintained during the infection) according to the experimental design and were either stained for analyzing the inclusion development or prepared for Western blot analysis.

### EGFR inhibitor treatment

Erlotinib and Cetuximab treatments were used for EGFR inhibition. The cells were pretreated with Erlotinib (25 μM) for 2 h and then infected with *C. trachomatis*. For Cetuximab treatment, cells were treated with 20 μg/ml drug for 2 h in DMEM +0.1% FBS followed by *C. trachomatis* infection. The inhibitor concentration was maintained during the infection. In certain experiments in which protein phosphorylation was investigated (Figure 1B, D, F, G, Figures 3, 4, and Additional file 1: Figure S2) the cells were serum starved overnight before drug treatment and *C. trachomatis* infection. DMSO was used as the vehicle control for Erlotinib and IgG was used as control treatment for Cetuximab. Infected samples were used either for Western blotting or confocal imaging as described below. In another set of experiments the cells were infected with *C. trachomatis* followed by addition of Erlotinib at 2.5, 5 and 18 hpi. The total time for *C. trachomatis* infection was 24 h after which the samples were used for Western blotting or confocal imaging. *Control experiments.* Chlamydial EBs were mixed with complete DMEM (DMEM + 10% FBS) containing either 25 μM Erlotinib, 20 μg/ml Cetuximab or DMSO and incubated for 2.5 h at 37°C, centrifuged at 16,000 rpm at 4°C for 30 minutes. EBs’ pellets were washed and resuspended in SPG buffer and used for subsequent HeLa cell infection. At 24 hpi cells were lysed for Western blotting of chlamydial Hsp60 antigen.

### Inside out staining

Differential staining of external and internalized bacteria was performed as described previously and using three independent studies [19]. Briefly, cells were grown overnight in 2-well Lab-Tek chamber slides and treated with either EGFR inhibitors or siRNA as described above and subsequently infected with *Chlamydia* for 2.5 h at 37°C to allow for bacterial attachment and internalization. For blocking EGFR with Cetuximab, cells were preincubated with Cetuximab or control IgG for 2 h before addition of bacteria. Infected cells were washed five times in PBS and fixed in 1% paraformaldehyde (PFA). After fixation, cells were blocked in 5% BSA for 1 h and then incubated with FITC-conjugated antibody against chlamydial EBs for 1 h to stain external EBs. Cells were then permeabilized with 0.1% Triton X-100, blocked again, and incubated with antibody against chlamydial LPS followed by incubation with rhodamine-conjugated anti-goat antibody to stain intracellular and extracellular EBs. Imaging was performed using confocal microscopy (Carl Zeiss, Germany). The quantification for the inside out experiments was performed manually based on the number of attached EBs observed per infected cell. The statistical analysis was based on imaging data collected from fifteen fields containing 3–10 cells per field as described below under “*Image acquisition and statistical analysis*”.

### Immunofluorescence

Cells were infected with *C. trachomatis* as described above. The cells were washed 5 times with PBS and fixed at either 2.5 hpi or 24 hpi with 4% PFA for 10 min and blocked with 5% BSA for 1 h. After washing with TBS (50 mM Tris.HCl, pH 7.4 and 150 mM NaCl) the cells were permeabilized for 15 min with 0.1% TritonX100 and again washed with TBS followed by incubation with the indicated primary antibodies overnight. The cells were washed three times (10 min each) with TBS and incubated with appropriate secondary antibodies and Alexa Fluor 488-phalloidin (1:40 dilution in PBS) for 1 h. After repeated washings, the coverslips were mounted and analyzed using Zeiss LSM 510 or 710 laser scanning confocal microscope. For the studies shown in Figure 1A, *C. trachomatis* inclusions were stained using the Pathfinder Chlamydia Culture Confirmation System (BioRad, Hercules, CA, USA). At 24 h post *C. trachomatis* infection, cells were fixed with methanol for 10 min at room temperature and stained with the FITC conjugated pathfinder anti-chlamydial mAb according to the manufacturer’s protocol (Bio-Rad, Hercules, CA). For the co-localization studies, 30,000 HeLa cells were seeded into EZ slide 4-well glass (Millipore, Temecula, CA, USA) and infected with *C. trachomatis* as described above for 2.5 h the next day. Cells were fixed 24 h later in 4% formaldehyde in PBS for 15 min, permeabilized in 0.1% Triton X-100 for 10 min, and blocked in 1% BSA for 1 h at room temperature. Then the cells were incubated with anti-EGFR rabbit mAb (Alexa Fluor 594 conjugate, Cell Signaling, Danvers, MA, USA) overnight at 4°C, followed by incubation with Alexa Fluor 488 Phalloidin (Invitrogen, Grand Island, NY, USA) for 1 h at room temperature. The slides were mounted with Fluoromount mounting medium (Sigma Aldrich, St. Louis, MO, USA), sealed, and examined using Zeiss 710 laser scanning confocal microscope.

### Intracellular calcium staining

Cells were washed with calcium free incomplete DMEM and incubated with 2 μM Fluo-4 AM diluted in Ca^2++^ free incomplete DMEM at 37°C for 30 min. The cells were then washed with Ca^2++^ free HBSS and analyzed for calcium levels using Olympus IX71 fluorescence microscope.

### BAPTA/AM and Ionomycin treatment

Monolayers of HeLa cells were washed with PBS and replaced with calcium free DMEM +1% FBS and pretreated for 1 h with BAPTA/AM (15 μM) followed by *C. trachomatis* infection for 24 h. BAPTA/AM concentration was maintained during the *C. trachomatis* infection. The control cells were treated with DMSO (<0.1%) followed by 24 h of *C. trachomatis* infection. In another set of experiments, HeLa cells were infected with *C. trachomatis* followed by addition of BAPTA/AM (15 μM) or DMSO at 2 or 5 hpi. The total time for *C. trachomatis* infection was 24 h. To induce mobilization of calcium from intracellular stores to the cytoplasm, cultured cells were pretreated for 1 h with 1 μg/ml Ionomycin before chlamydial infection. *C. trachomatis*-infected cells were washed three times with PBS after 24 hpi and processed for immunofluorescence as described above.

### Cell proliferation assay

To ensure that the dose of Erlotinib (25 μM) provided maximal inhibition without affecting cell viability, MTT assays were performed. HeLa cells were cultured for 24 h, treated with 25 μM Erlotinib and incubated at 37°C, 5% CO_2_ for 24 h. After washing the cells, the procedure for cell viability assay was followed as per manufacturer’s instructions (Roche).

### Transmission electron microscopy

HeLa cells were infected with *C. trachomatis* as described above. Twenty-four hours post chlamydial infection the cells were washed with PBS and fixed with 2.5% glutaraldehyde in 0.1 N Millonig’s buffer (pH 7.2) for 1 h at room temperature. The cells were then washed and post-fixed for 1 h in 1% osmic acid in 0.1 N Millonig’s buffer followed by 1 h treatment with 1% uranyl acetate. A graded ethanol series (25%, 50%, 70-75%, 90-95% and 100%) was used to dehydrate the cells prior to embedding in Spurr’s resin. Thin sections were then cut with a Reichert ultracut E microtome and stained with 1% uranyl acetate and Reynold’s lead citrate solutions, followed by the analysis using 80 kV Tecnai Spirit BioTwin transmission electron microscope.

### Image acquisition and statistical analysis

Images of stained cells were acquired in a Z-series on a Zeiss LSM 710 AxioObserver Z.1 inverted laser scanning confocal microscope using a Zeiss Plan-Apochromat 63×/1.3 water-immersion objective with 3× digital zoom at the Wake Forest University Microscopic Imaging Core Facility and Confocal Microscopy Center. Lasers of 405 nm (25 mW diode), 488 nm (35 mW Argon laser), and 594 nm (2 mW He/Ne laser) were used to illuminate the samples and images were captured using a R6357 photomultiplier tube (Hamamatsu Photonics, Hamamatsu City, Japan) with a pixel dwell time of 0.79 μs. Final image magnification at the time of image capture was 1,890×, with each voxel representing 0.02 μm × 0.02 μm × 0.39 μm. A pinhole of 53.88 μm (~1 Airy unit for the red channel) was used for all color channels of all images. All images were captured at 2048 × 2048 pixels, saved in 8-bit .lsm image format, and converted to .tif format for analysis in the MacBiophotonics ImageJ package (McMaster University Biophotonics Facility, Hamilton, Ontario, CA). A median filter of 7 × 7 pixels was applied to EGFR images using Zeiss Zen 2011 Blue Edition (Carl Zeiss Microscopy GmbH, Göttingen, Germany) to reduce background noise. Co-localization of each image was determined using eight independent techniques. Standard overlays and intensity profile data were generated using Zen 2011, while six different co-localization analysis algorithms were performed using the JACoP [48] and Colocalization Colormap [47] plugins for ImageJ (National Institutes of Health, USA).

ImageJ was used for quantification of the Western blots, counting and estimation of chlamydial inclusion size and for the counting of the bound and internalized EBs (inside out studies). To define the chlamydial inclusion number and size at least fifteen random fields were analyzed for each result. The number of inclusions was calculated per 10^5^ cells and expressed as a percentage of the respective controls. Similarly, fifteen random fields (3–10 cells per field) were used for the inside out experiments. All results are presented as mean ± SEM. t-test was used for comparisons and calculating the level of significance using SigmaPlot version 12.0.

## Additional files

Additional file 1
**Control experiments to support studies in Figures** 1, 2, 3, 4, 5, 6 and **7**. Additional file 2
**Supplementary data to support the function of calcium in Ct infection.**
Additional file 3
**Description of methods used for co-localization analysis and additional co-localization figures.**

## Abbreviations

Akt: Protein kinase B
Ct: *Chlamydia trachomatis*
DMSO: Dimethyl sulfoxide
EB: Elementary body
EGF: Epidermal growth factor
EGFR: Epidermal growth factor receptor
Erk: Extracellular signal-regulated kinase
FGF: Fibroblast growth factor
FGFR: Fibroblast growth factor receptor
Hpi: Hours post infection
HPV: Human papillomavirus
HSP60: Heat shock protein 60
IgG: Immunoglobulin G
LPS: Lipopolysaccharide
mAb: monoclonal antibody
MEF: Mouse embryonic fibroblast
nMDP: normalized mean deviation product
PDGFR: Platelet-derived growth factor receptor
PI3K: Phosphatidylinositol-4,5-bisphosphate 3-kinase
PLC: Phospholipase C
RB: Reticulate body
siRNA: small interfering RNA
STAT: Signal transducer and activator of transcription
