# Supplementary material for: Activation of epidermal growth factor receptor is required for Chlamydia trachomatis development
Source: BMC Microbiol. 2014 Dec 4;14:277. doi: 10.1186/s12866-014-0277-4 (PMC4269859; doi:10.1186/s12866-014-0277-4)
Supplement: Additional file 1 — Control experiments to support studies in Figures 1, 2, 3, 4, 5, 6 and 7. [file 12866_2014_277_MOESM1_ESM.pdf]

Additional File 1. Control experiments to support studies in Figures 1-7.

Figure S1. Western blot analysis of EGFR phosphorylation with Erlotinib treatment. HeLa cells were treated with Erlotinib (15 μM, 25 μM) followed by Western blotting. Phosphorylation of EGFR was significantly reduced in comparison to the untreated cells. β-actin was used as loading control.

Figure S2. Western blot analysis of EGFR phosphorylation induced by Ct infection in the absence or presence of Erlotinib treatment. HeLa cells were treated with 25 μM Erlotinib for 2 h followed by the Ct infection for 2.5 h. Western blotting with pEGFR antibody showed an increase in EGFR phosphorylation upon Ct infection in the absence of Erlotinib but not in the presence of the drug. β-actin was used as loading control.

Figure S3. Western blot analysis of EGFR expression. HeLa cells were treated with EGFR siRNA followed by Western blotting. siRNA treatment significantly reduced the protein levels of EGFR by 70% in comparison to the control siRNA treated cells. β-actin was used as loading control. This study also confirmed that expression of PDGFRβ remained unaffected in EGFR siRNA treated cells.

Figure S4. Western blot analysis of chlamydial Hsp60. HeLa cells were treated with Erlotinib (25 μM) followed by Ct infection for 24 h. Western blotting with anti-chlamydial Hsp60 antibody showed marked decrease in the Hsp60 antigen in the Erlotinib treated cells. β-actin was used as loading control.

Figure S5. Western blot analysis to test the viability of EBs treated with Erlotinib and Cetuximab. HeLa cells were pretreated with Erlotinib (25 μM) or Cetuximab (20 μg/ml) for 2.5 h followed by Ct infection for 24 h. Western blotting with anti-chlamydial Hsp60 antibody showed no difference in the chlamydial Hsp60 antigen load between the cells infected with either drug-treated or untreated EBs. β-actin was used as loading control.

Figure S6. Cell viability assay. HeLa cells were treated with 25 μM of Erlotinib for 24 h followed by the MTT assay (Roche). Erlotinib treatment did not affect the viability of HeLa cells.

Figure S7. Western blot analysis of PDGFRβ expression. HeLa cells were treated with PDGFRβ siRNA followed by Western blotting. siRNA treatment significantly reduced the protein levels of PDGFRβ in comparison to the control siRNA treated cells. β-actin was used as loading control. It was also confirmed that expression of EGFR remained unaffected in PDGFRβ siRNA treated cells.

Figure S8. Western blot analysis of EGFR in MEFs EGFR+/+ and MEFs EGFR−/−. EGFR was expressed only in MEFs EGFR+/+. β-actin was used as loading control.

Figure S9. Western blot analysis of chlamydial Hsp60. HeLa cells were infected with Ct and then treated with Erlotinib (25 μM) at either 2.5 hpi or 18 hpi. The total time for Ct infection was 24 h followed by Western blotting with anti-chlamydial Hsp60 antibody. Significant decrease in the Hsp60 was observed in cells treated with Erlotinib at 2.5 hours post Ct infection but not in cells treated with Erlotinb at 18 hpi. β-actin was used as loading control.

Figure S1

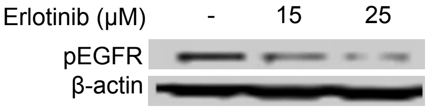

Figure S2

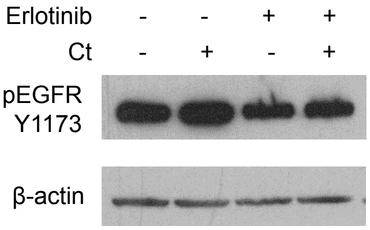

Figure S3

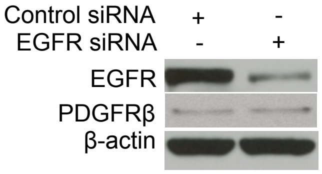

Figure S4

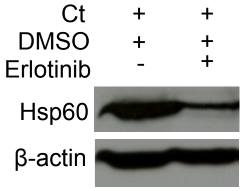

Figure S5

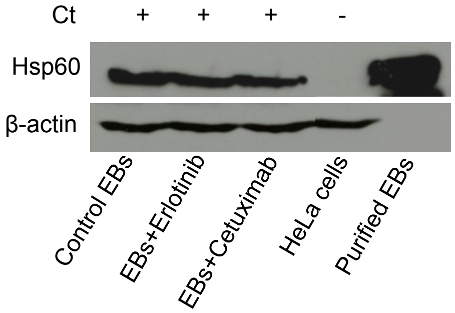

Figure S6

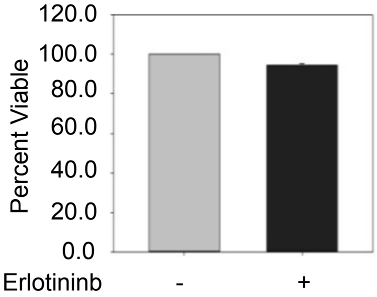

Figure S7

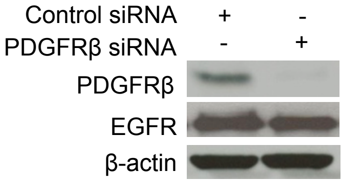

Figure S8

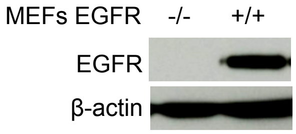

Figure S9

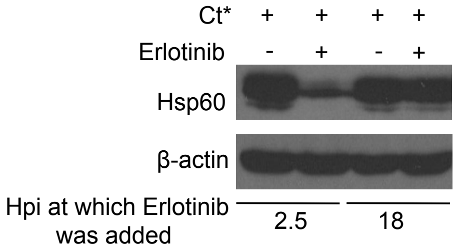

*Total time for Ct infection was 24 h
