# Supplementary material for: Activation of epidermal growth factor receptor is required for Chlamydia trachomatis development
Source: BMC Microbiol. 2014 Dec 4;14:277. doi: 10.1186/s12866-014-0277-4 (PMC4269859; doi:10.1186/s12866-014-0277-4)

Additional File 2. Supplementary data to support the function of calcium in Ct infection.

Figure S10. (A-D) HeLa cells were infected with Ct for 24 h. In panels C & D, EGFR inhibitor Erlotinib was added at 2.5 and 5 hpi respectively. Under all conditions the total time for Ct infection was 24 h and cells were stained with Fluo-4 AM for visualization of calcium ($Ca^{2+)}$ by fluorescence microscopy. (E) The fluorescence intensity of calcium staining shown in panels A-D was quantified using Image J. Note the weak calcium signal in EGFR inhibited cells ($P<0.05$) in comparison with cells infected with Ct in the absence of Erlotinib.

Figure S11. Effect of Ionomycin treatment on inclusion formation. (A) HeLa cells were treated with Ionomycin (1 µg/ml) for 1 h and stained with Fura-2/AM for visualization of calcium (Ca^{2+}) by fluorescence microscopy. Increased intracellular free calcium was observed with Ionomycin treatment. (B) HeLa cells treated with EGFR siRNA with or without one hour pre-treatment with Ionomycin (1 µg/ml) were infected with Ct for 24 h and fixed, processed for confocal microscopy to compare the inclusion formation in comparison to the Ct-infected control cells. F-actin was detected with Alexa Fluor 488-phalloidin (green) and chlamydial inclusions were detected using anti-chlamydial LPS antibody (red). In comparison to control, small inclusions were formed in the EGFR depleted cells and no significant difference was observed between the inclusions formed in EGFR siRNA treated cells with or without Ionomycin treatment. Scale bar - 10 µm.

Figure S12. F-actin staining in HeLa cells. F-actin staining was performed with Alexa Fluor 488-phalloidin (green) in HeLa cells treated with Erlotinib or BAPTA/AM. F-actin staining for untreated HeLa cells is also shown.

Figure S13. Inclusion development in PDGFR siRNA treated cells. Hela cells treated with control siRNA or PDGFR siRNA were infected with Ct for 24 h, fixed, and processed for confocal microscopy. F-actin was detected with Alexa Fluor 488-phalloidin (green) and chlamydial inclusions were detected using anti-chlamydial LPS antibody (red). Note that silencing PDGFR did not affect the size of the inclusion.

Figure S10

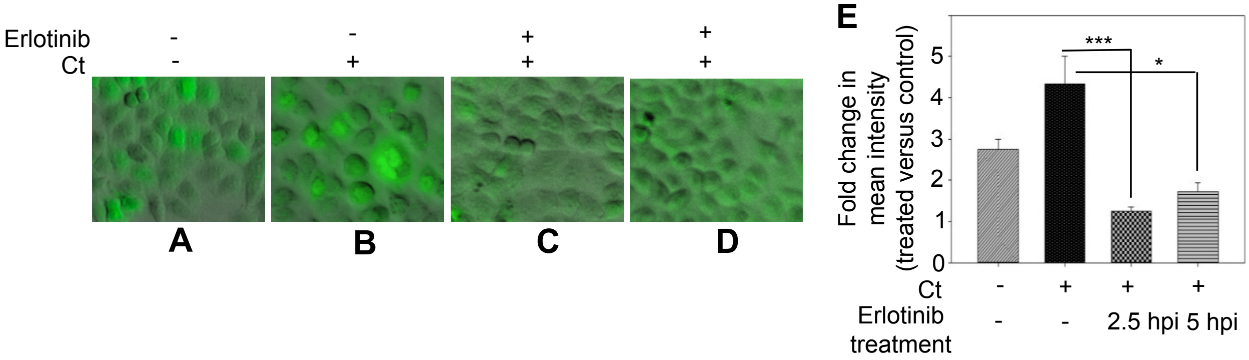

Figure S11

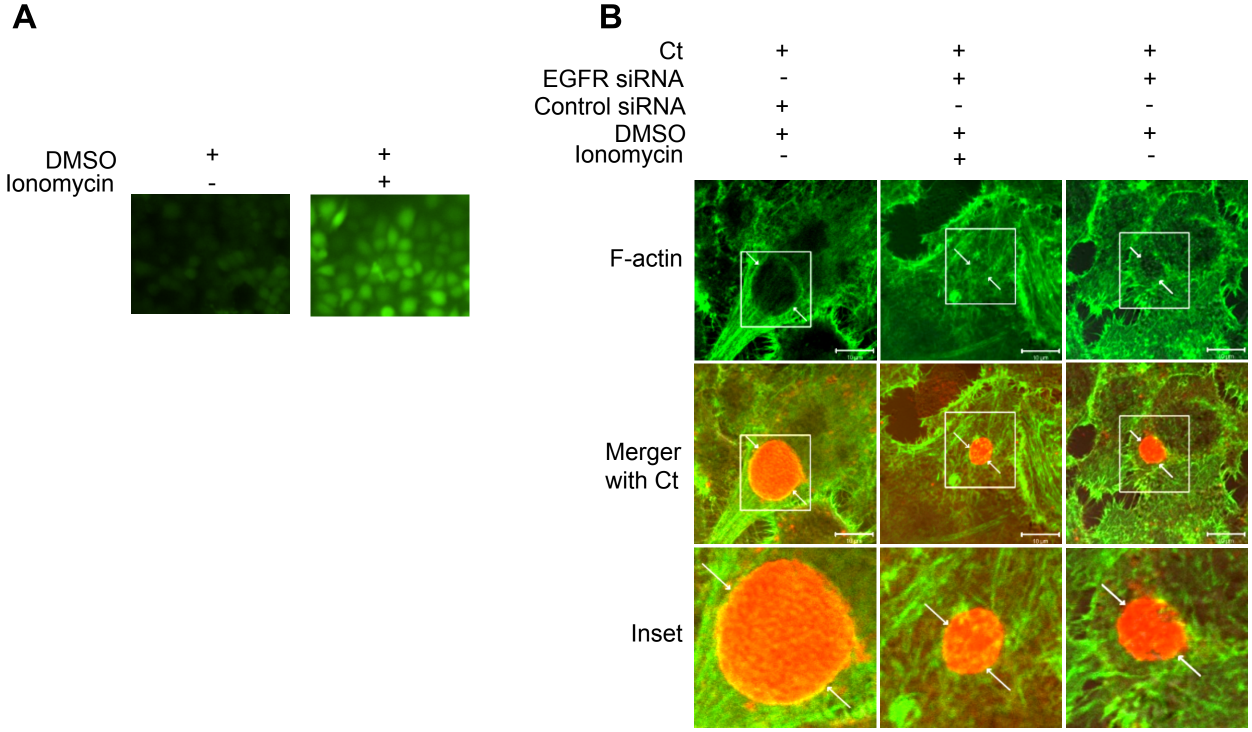

Figure S12

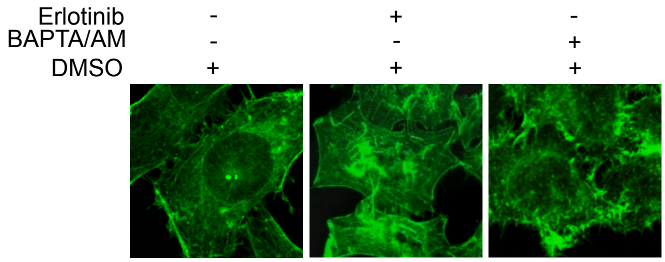

Figure S13

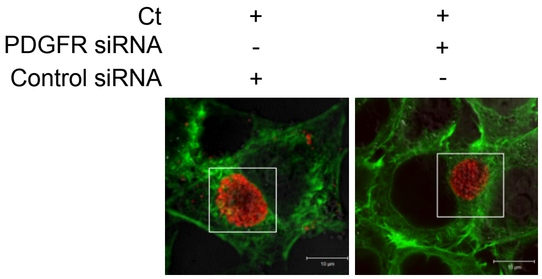

Supplement: Additional file 2 — Supplementary data to support the function of calcium in Ct infection. [file 12866_2014_277_MOESM2_ESM.pdf]
