# Supplementary material for: Activation of epidermal growth factor receptor is required for Chlamydia trachomatis development
Source: BMC Microbiol. 2014 Dec 4;14:277. doi: 10.1186/s12866-014-0277-4 (PMC4269859; doi:10.1186/s12866-014-0277-4)
Supplement: Additional file 3 — Description of methods used for co-localization analysis and additional co-localization figures. [file 12866_2014_277_MOESM3_ESM.pdf]

Additional File 3. Description of methods used for co-localization analysis and additional co-localization figures.

Methods. In order to determine the interaction between EGFR and F-actin, four independent methods were used for analysis of imaging data. First, Cytofluorograms [48] are generated to demonstrate the relationship between intensities of each color channel (EGFR - red; F-actin - green) at each pixel - a positive slope greater than 0.5 and a tight scatter of data points indicates co-localization of EGFR with F-actin. The increased slope of the inclusion cytofluorogram indicates higher colocalization of EGFR and F-actin than the non-inclusion image. This information was then utilized to determine the Pearson's Coefficient. Second, Li's Intensity Correlation Analysis (ICA) [48] is performed to gain further insight into the degree of co-localization. It calculates the difference from the mean channel intensity of each color at each pixel. Li's ICA analysis indicates noise-corrupted co-localization of EGFR and F-actin in the inclusion area images as indicated by the points mostly falling on the positive side of the x-axis. The non-inclusion area image is found to have almost no correlation between EGFR and F-actin as indicated by the points falling roughly equally along the positive and negative sides of the x-axis. Li's ICA was also used here to determine Li's intensity correlation quotient (ICQ). Third, Van Steensel's CCA is performed by shifting one color channel in the x-direction pixel per pixel relative to the other channel and calculating the respective Pearson's Coefficient. The resulting Pearson's Coefficients are then plotted as a function of the pixel shift. Bell-shaped data in Van Steensel's CCA indicates co-localization and trough-shaped data indicates exclusion. Van Steensel's CCF analysis shows co-localization of EGFR and F-actin with unequal signal brightness in the inclusion area images and very weak, noise-corrupted partial overlap in the non-inclusion area images. Finally, Costes' randomization algorithm [48] is performed to rule out the possibility that the observed co-localization of EGFR and F-actin is attributable simply to random noise. Costes' randomization algorithm generates a number of images populated by various amounts of noise in each color channel and calculates the Pearson's coefficient for each one (represented by the data points along the blue line). Costes' randomization algorithm shows that the Pearson's coefficients of both the inclusion area images and the non-inclusion area image are due to signal rather than noise. Additional algorithm used in Figure 8 and Additional file 3: FigureS15-S20: The normalized mean deviation product (nMDP) [47] is calculated for each pixel in the image to identify regions of intense co-localization or exclusion within the image. The nMDP analysis operates on the same basic principles as Li's ICA, except that pixel position information is maintained, allowing the user to visualize where in the image the algorithm has identified co-localization or exclusion. This algorithm is also used here to determine the index of correlation (Icorr). Manders' coefficients [48] are calculated to provide further insight into the relationship between EGFR and F-actin within each image. The EGFR/F-actin Manders' coefficient is a measure of the percentage of EGFR signal that is co-localized with F-actin signal. Conversely, the F-actin/EGFR Manders' coefficient is a measure of the percentage of F-actin signal that is co-localized with EGFR signal.

Figure S14-S20. Comparison of cytofluorogram, Li's ICA, Van Steensel's CCF, and Costes' randomization analyses for EGFR and F-actin channels of representative inclusion and non-inclusion area images and additional algorithms used to generate data in Figure 8D.

Figure S14 (Supplementary for Figure 8A-C)

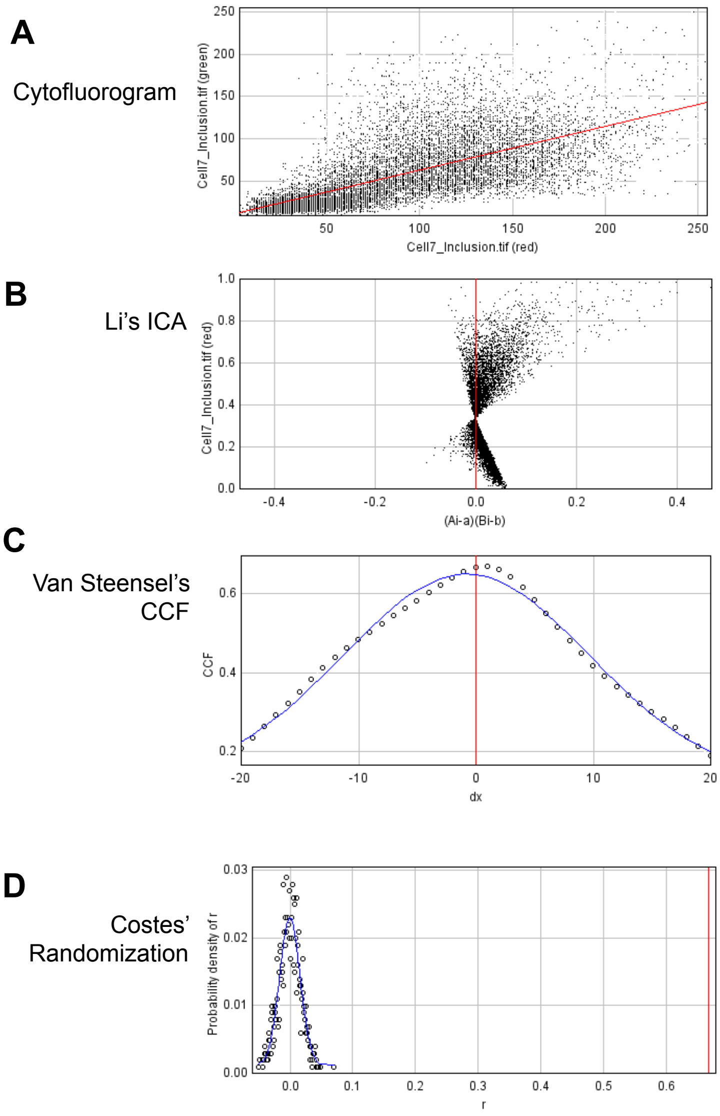

Colocalization Parameter	Range	Value	% Colocalization
Pearson's Coefficient	-1:1	0.669	66.9
Icorr	-1:1	0.801	80.1
Manders' EGFR/F-actin<br>Manders' F-actin/EGFR	0:1	0.612<br>0.813	61.2<br>81.3
Li's ICQ	-0.5:0.5	0.294	58.8

Figures S15-S20 (Supplementary for Figure 8D)

Figure S15 (Ct infected HeLa cell #1)

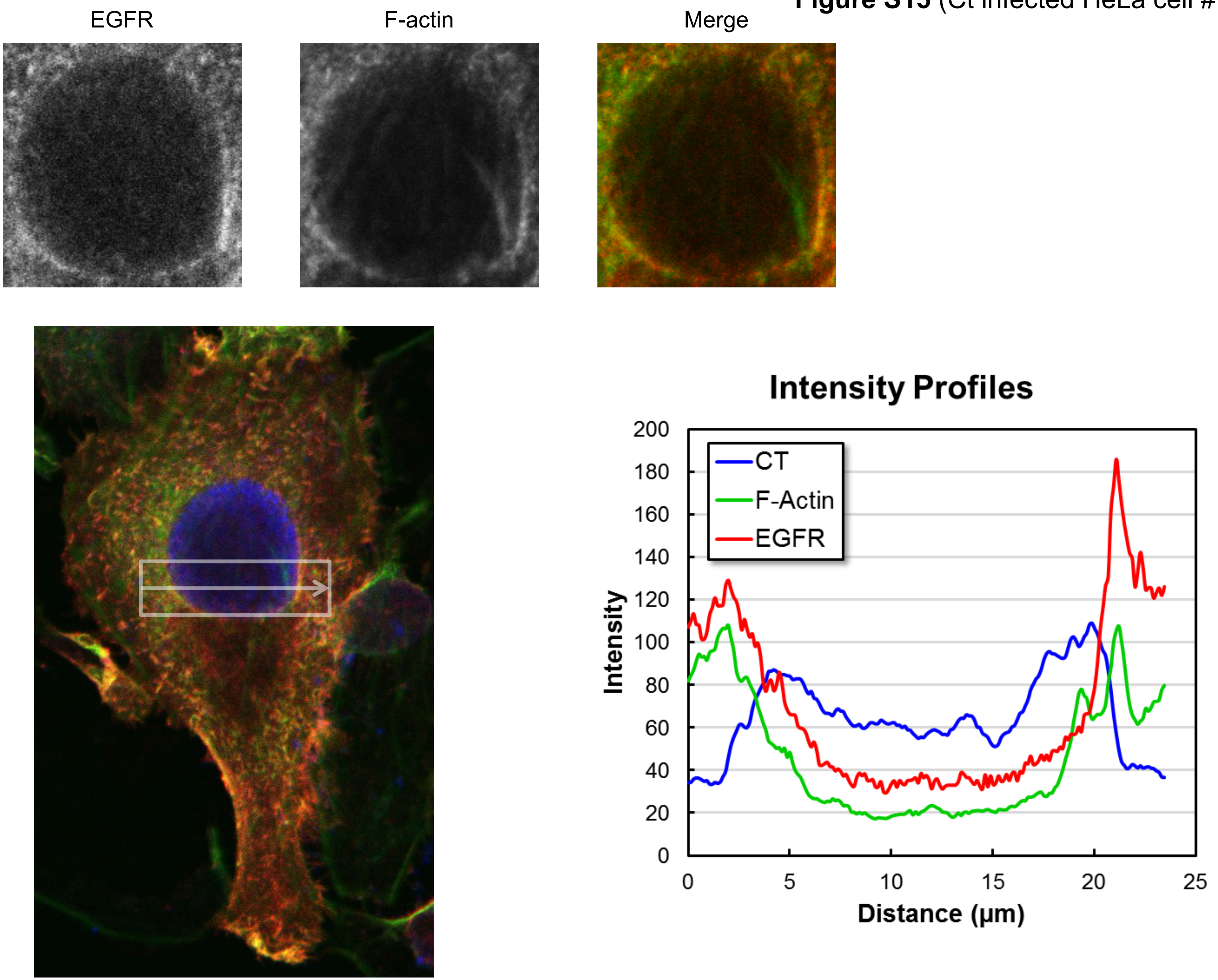

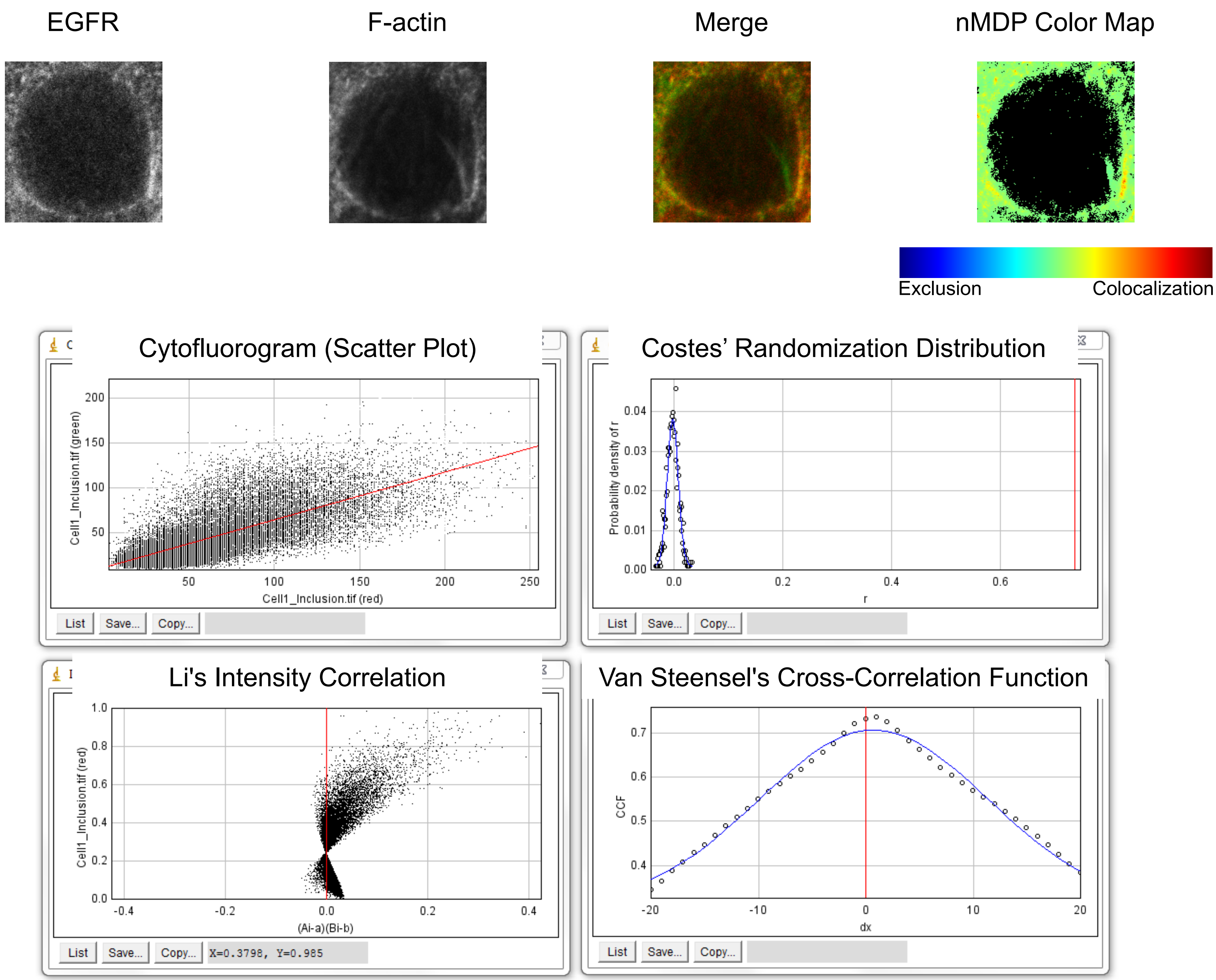
EGFR
F-actin
Merge
nMDP Color Map
Exclusion
Colocalization
Cytofluorogram (Scatter Plot)
Cell1_Inclusion.tif (green)
Cell1_Inclusion.tif (red)
Costes' Randomization Distribution
Probability density of r
r
Li's Intensity Correlation
Cell1_Inclusion.tif (red)
(Ai-a)(Bi-b)
X=0.3798, Y=0.985
Van Steensel's Cross-Correlation Function
CCF
dx
List
Save...
Copy...

Colocalization Parameter	Range	Value	% Colocalization
Pearson's Coefficient	-1:1	0.734	73.4
Icorr	-1:1	0.840	84.0
Manders' EGFR/F-actin<br>Manders' F-actin/EGFR	0:1	0.714<br>0.773	71.4<br>77.3
Li's ICQ	-0.5:0.5	0.334	66.8

Figure S16 (Ct infected HeLa cell #2)

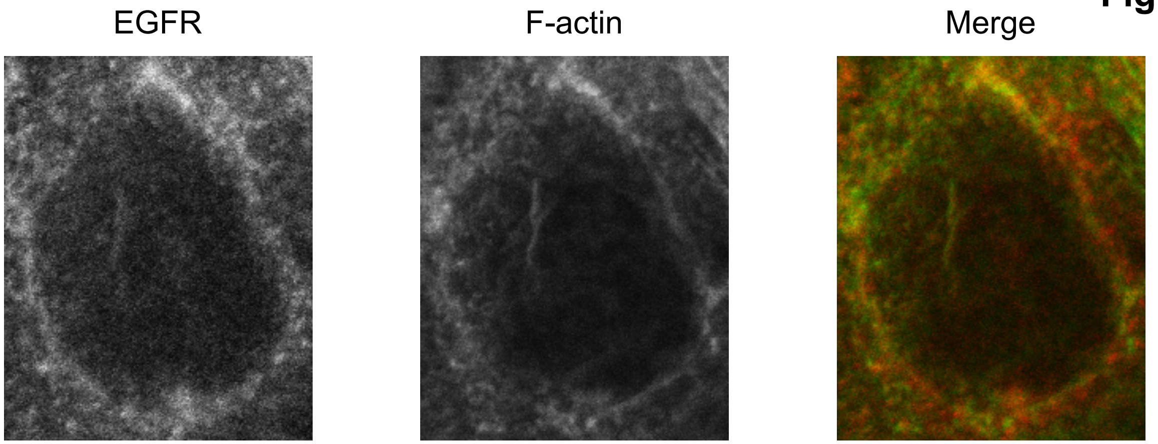

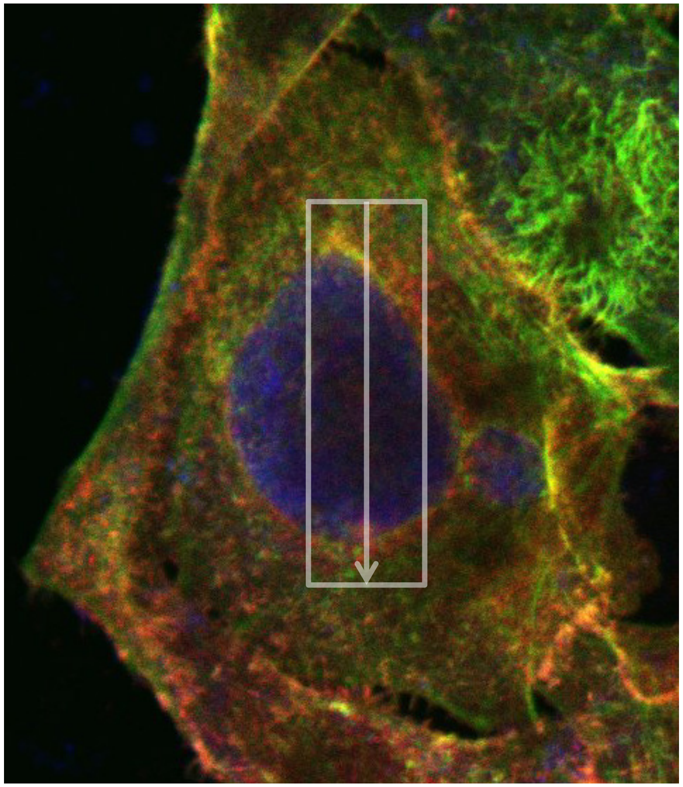

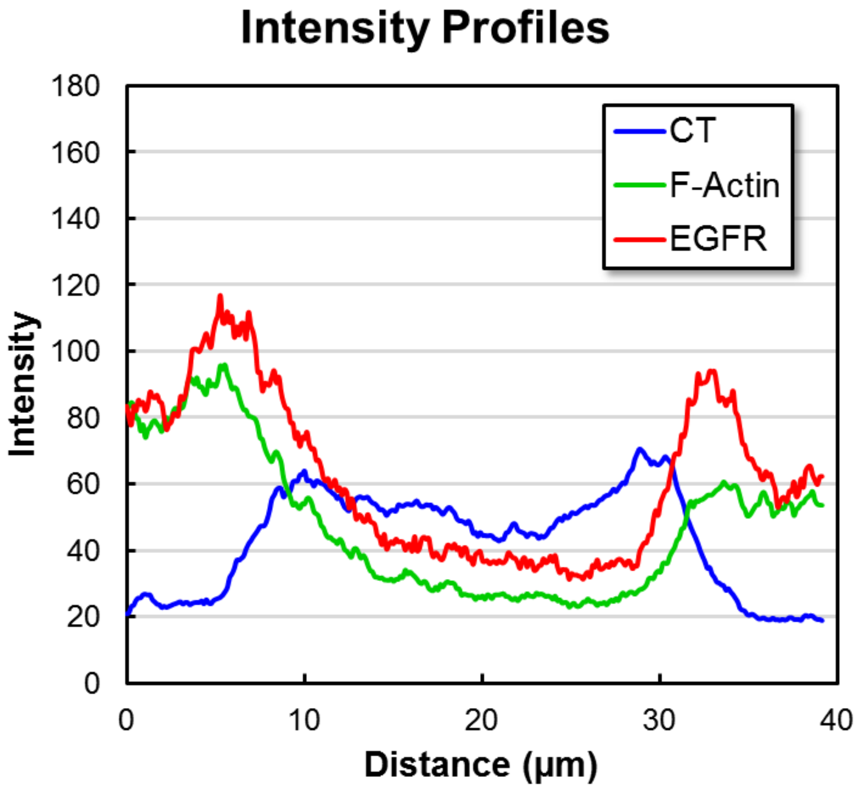

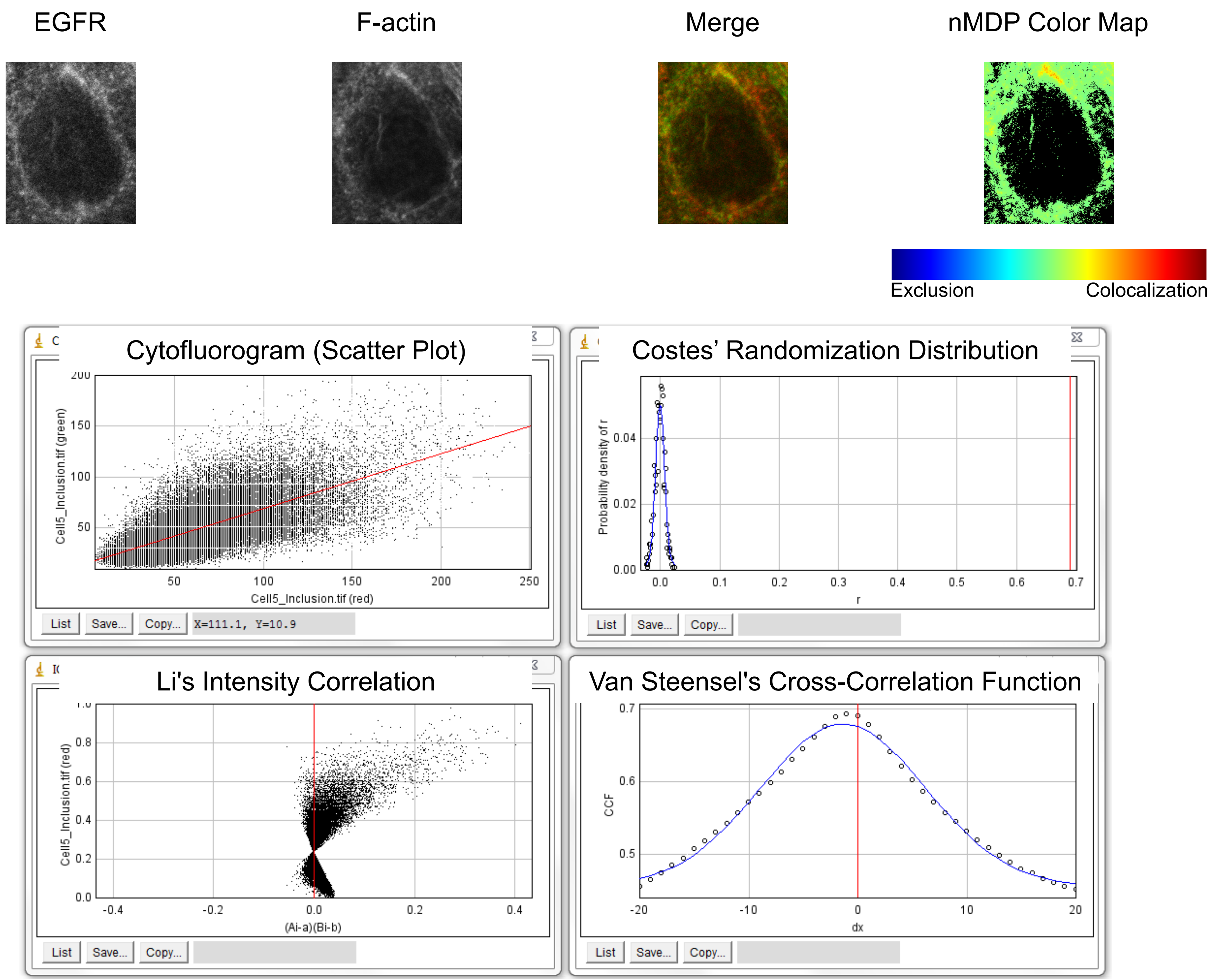
EGFR
F-actin
Merge
nMDP Color Map
Exclusion
Colocalization
Cytofluorogram (Scatter Plot)
Cell5_Inclusion.tif (green)
Cell5_Inclusion.tif (red)
List
Save...
Copy...
X=111.1, Y=10.9
Costes' Randomization Distribution
Probability density of r
r
Li's Intensity Correlation
Cell5_Inclusion.tif (red)
(Ai-a)(Bi-b)
Van Steensel's Cross-Correlation Function
CCF
dx

Colocalization Parameter	Range	Value	% Colocalization
Pearson's Coefficient	-1:1	0.690	69.0
Icorr	-1:1	0.786	78.6
Manders' EGFR/F-actin<br>Manders' F-actin/EGFR	0:1	0.683<br>0.724	68.3<br>72.4
Li's ICQ	-0.5:0.5	0.279	55.8

Figure S17 (Ct infected HeLa cell #3)

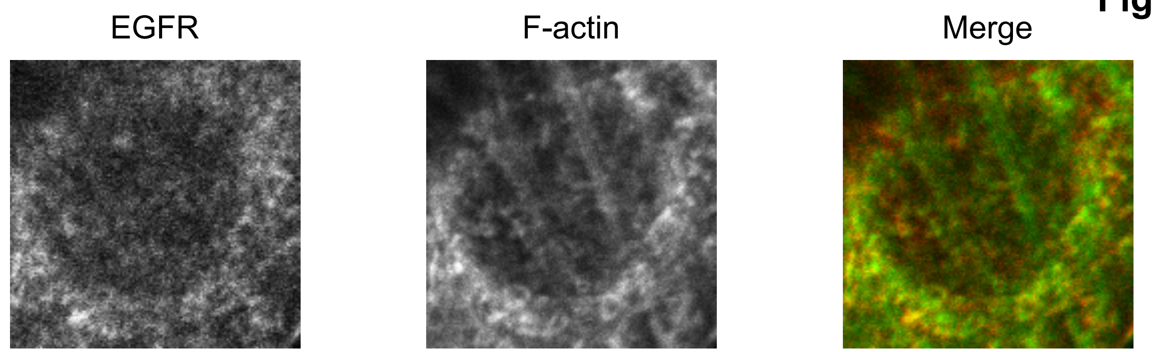

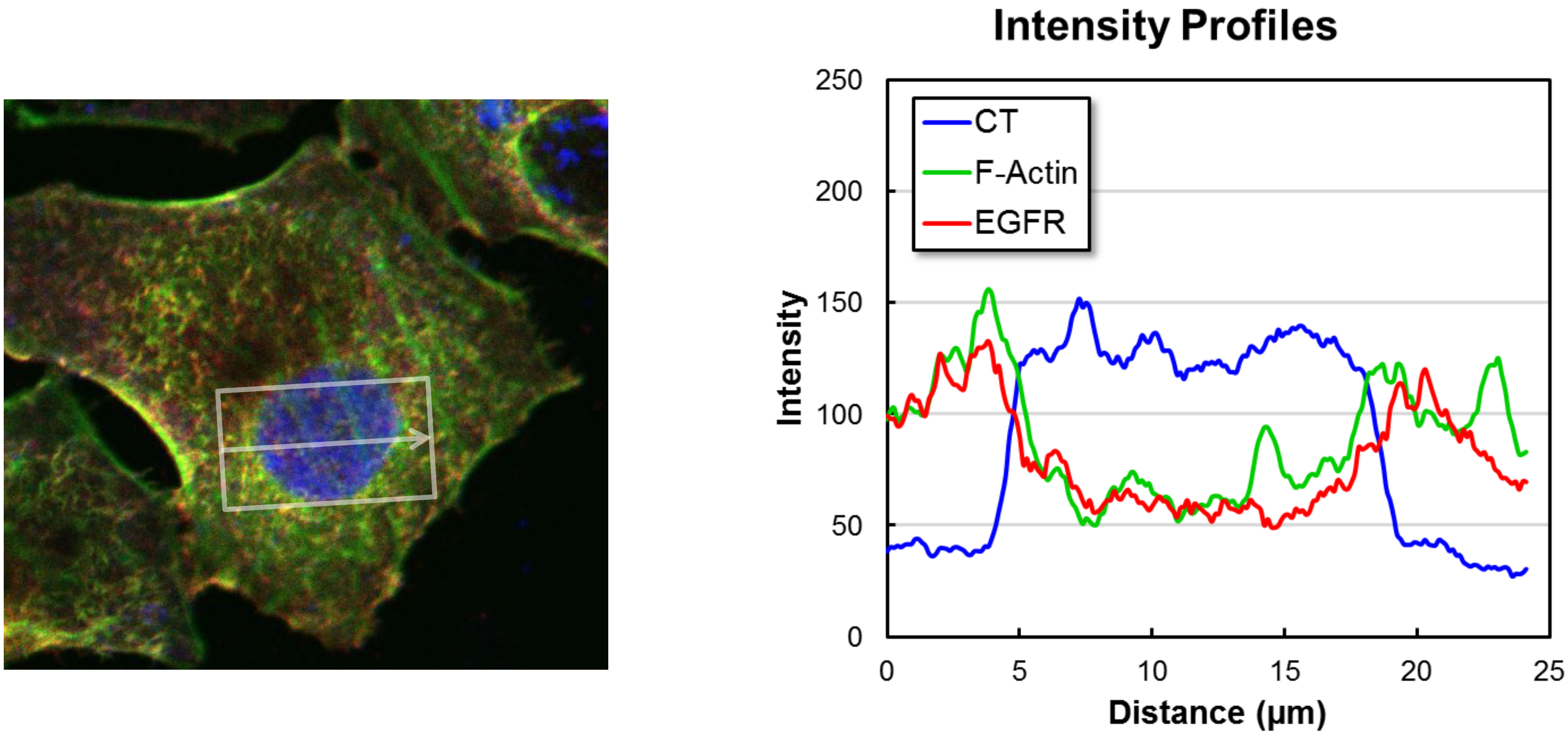

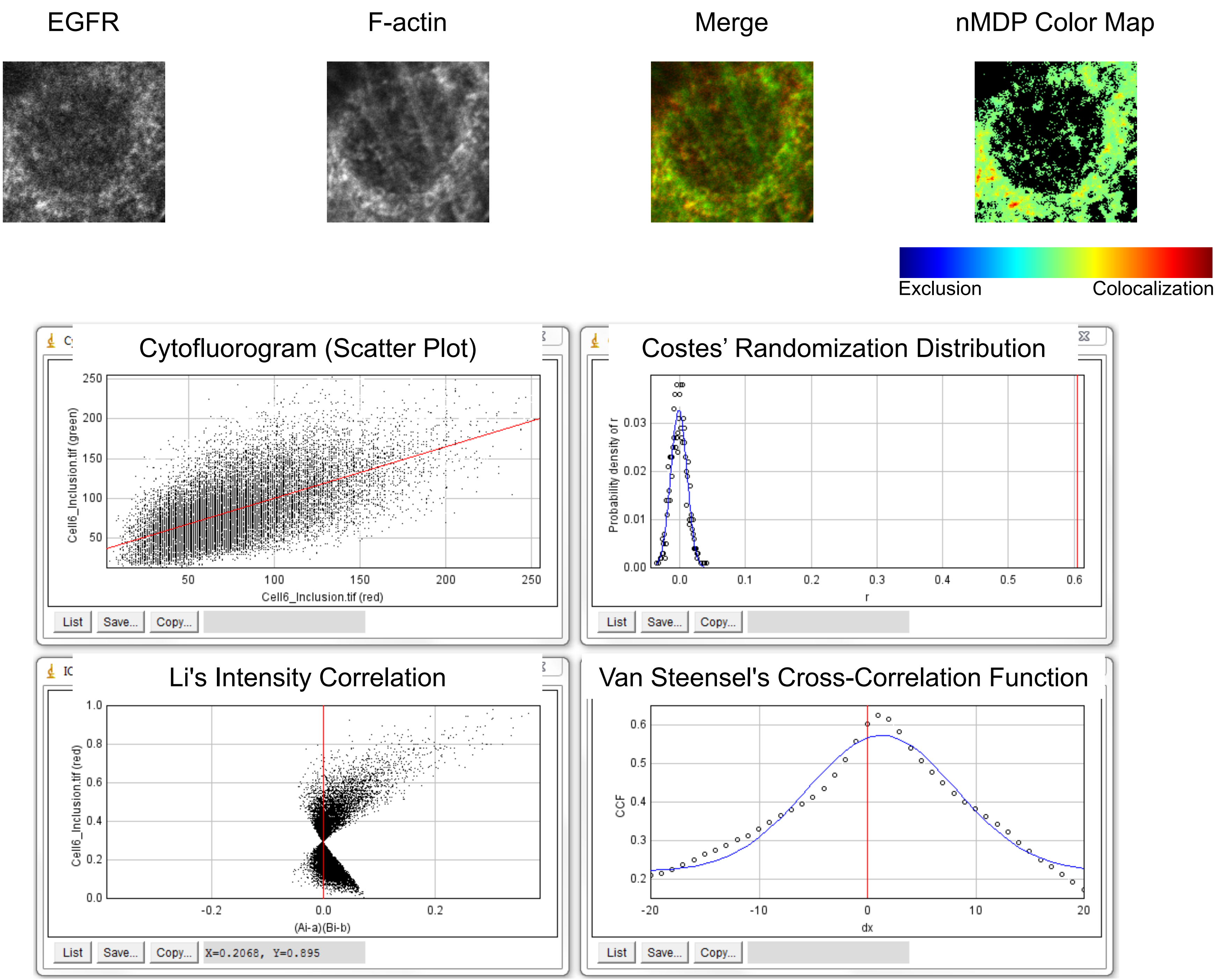
EGFR
F-actin
Merge
nMDP Color Map
Exclusion
Colocalization
Cytofluorogram (Scatter Plot)
Cell6_Inclusion.tif (green)
Cell6_Inclusion.tif (red)
Costes' Randomization Distribution
Probability density of r
r
Li's Intensity Correlation
Cell6_Inclusion.tif (red)
(Ai-a)(Bi-b)
X=0.2068, Y=0.895
Van Steensel's Cross-Correlation Function
CCF
dx
List
Save...
Copy...

Colocalization Parameter	Range	Value	% Colocalization
Pearson's Coefficient	-1:1	0.604	60.4
Icorr	-1:1	0.721	72.1
Manders' EGFR/F-actin<br>Manders' F-actin/EGFR	0:1	0.669<br>0.645	66.9<br>64.5
Li's ICQ	-0.5:0.5	0.217	43.4

Figure S18 (uninfected HeLa cell #1)

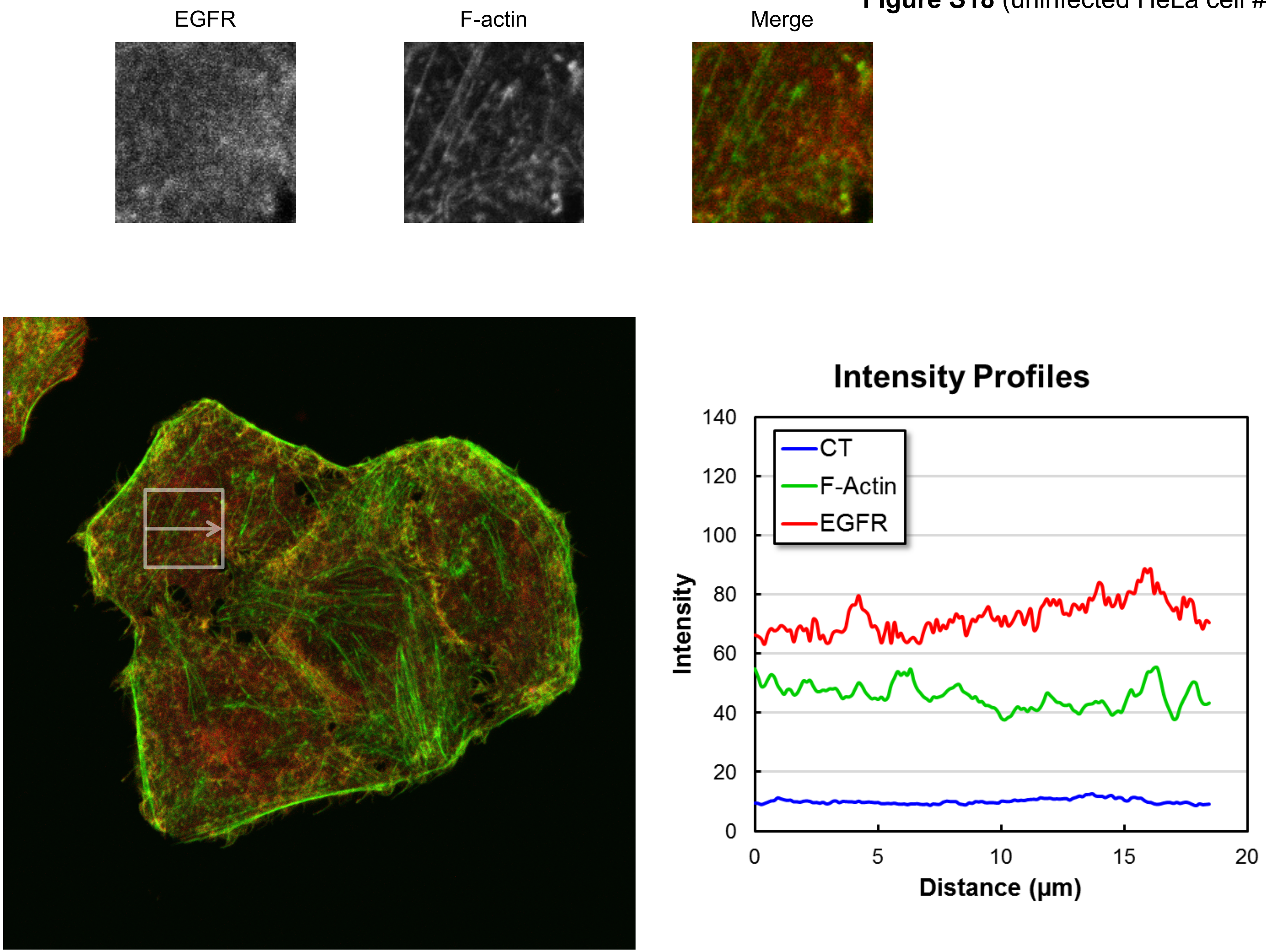

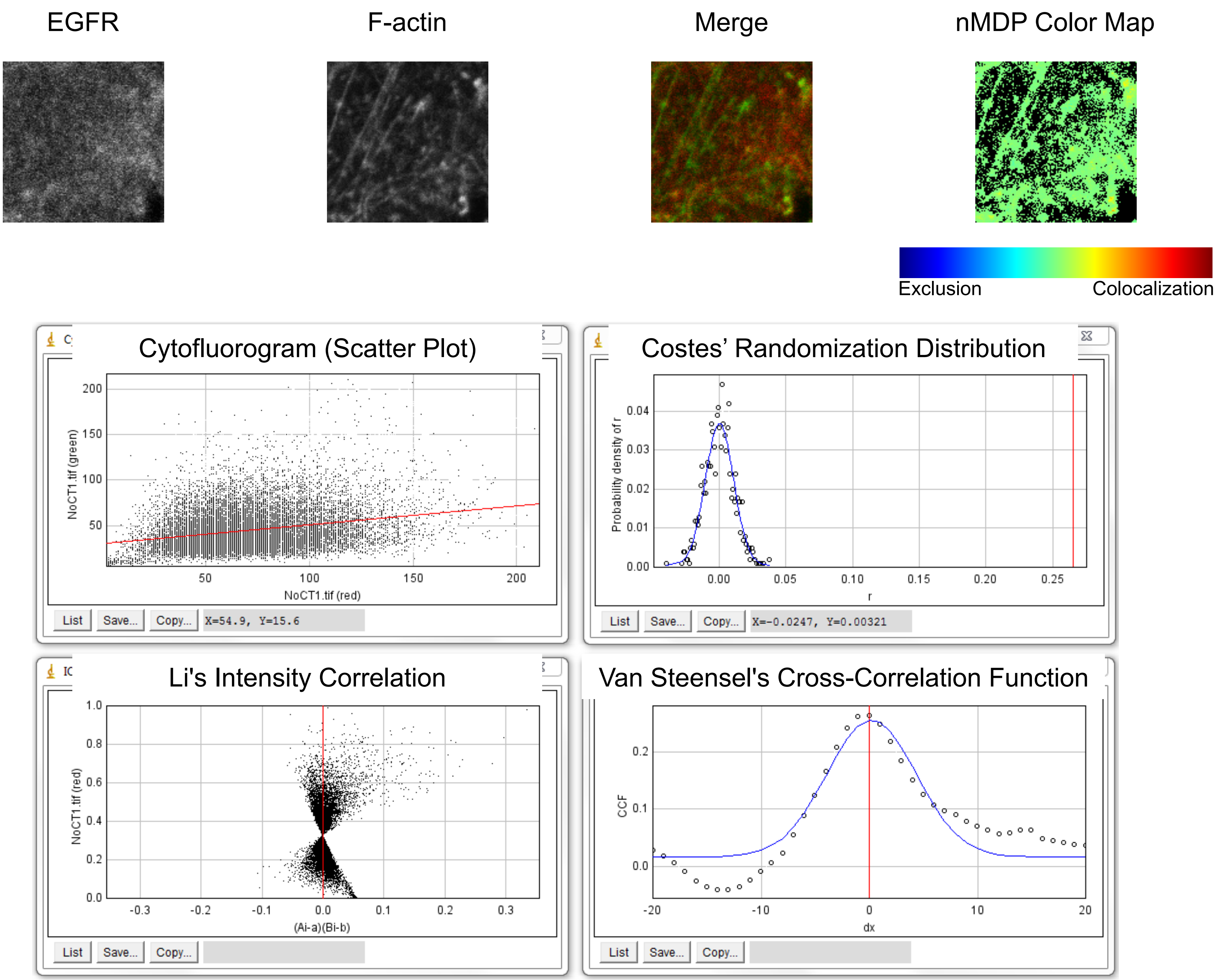
EGFR
F-actin
Merge
nMDP Color Map
Exclusion
Colocalization
Cytofluorogram (Scatter Plot)
NoCT1.tif (green)
NoCT1.tif (red)
List
Save...
Copy...
X=54.9, Y=15.6
Costes' Randomization Distribution
Probability density of r
r
List
Save...
Copy...
X=-0.0247, Y=0.00321
Li's Intensity Correlation
NoCT1.tif (red)
(Ai-a)(Bi-b)
List
Save...
Copy...
Van Steensel's Cross-Correlation Function
CCF
dx
List
Save...
Copy...

Colocalization Parameter	Range	Value	% Colocalization
Pearson's Coefficient	-1:1	0.265	26.5
Icorr	-1:1	0.584	58.4
Manders' EGFR/F-actin<br>Manders' F-actin/EGFR	0:1	0.366<br>0.525	36.6<br>52.5
Li's ICQ	-0.5:0.5	0.082	16.4

Figure S19 (uninfected HeLa cell #2)

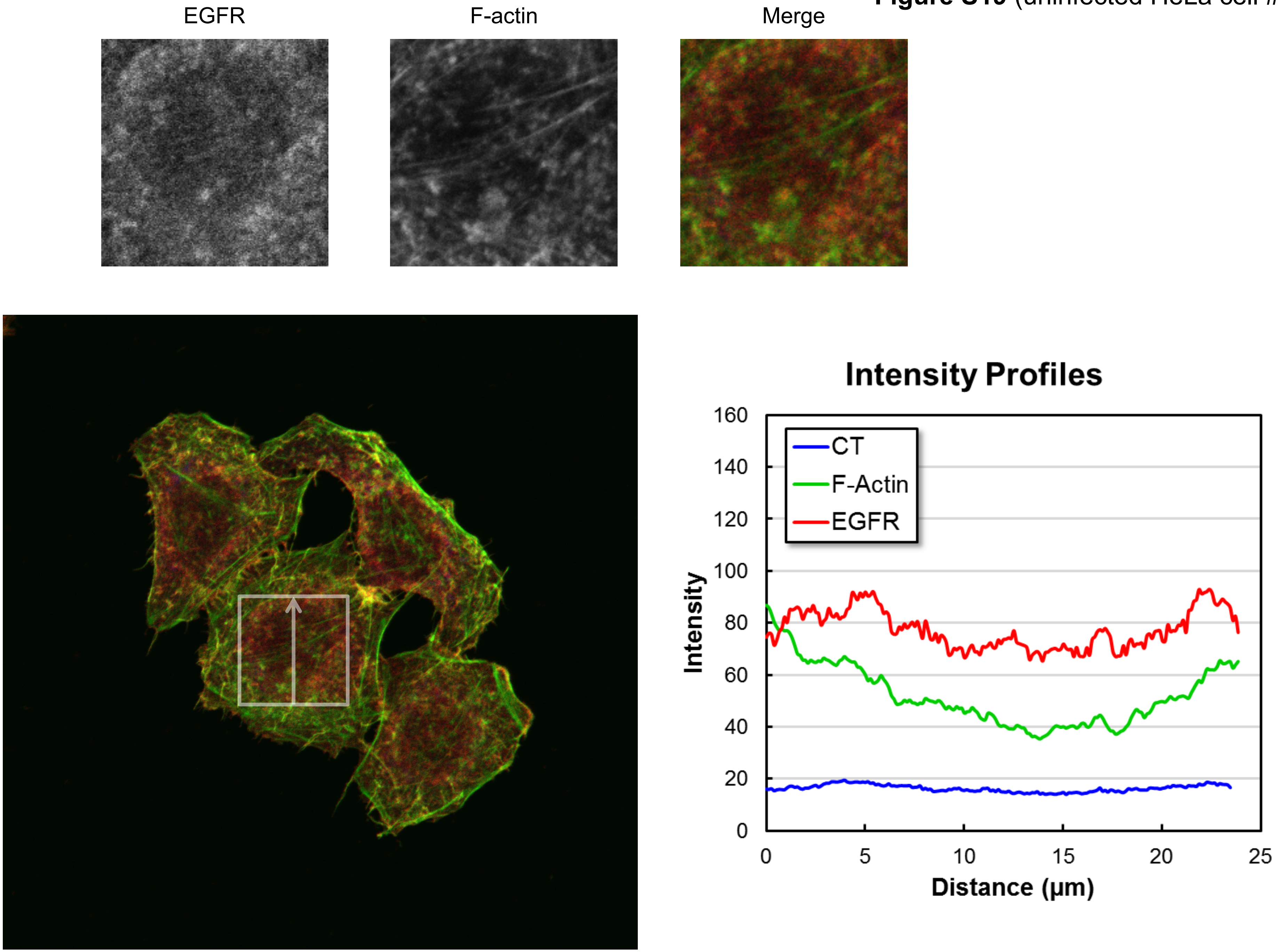

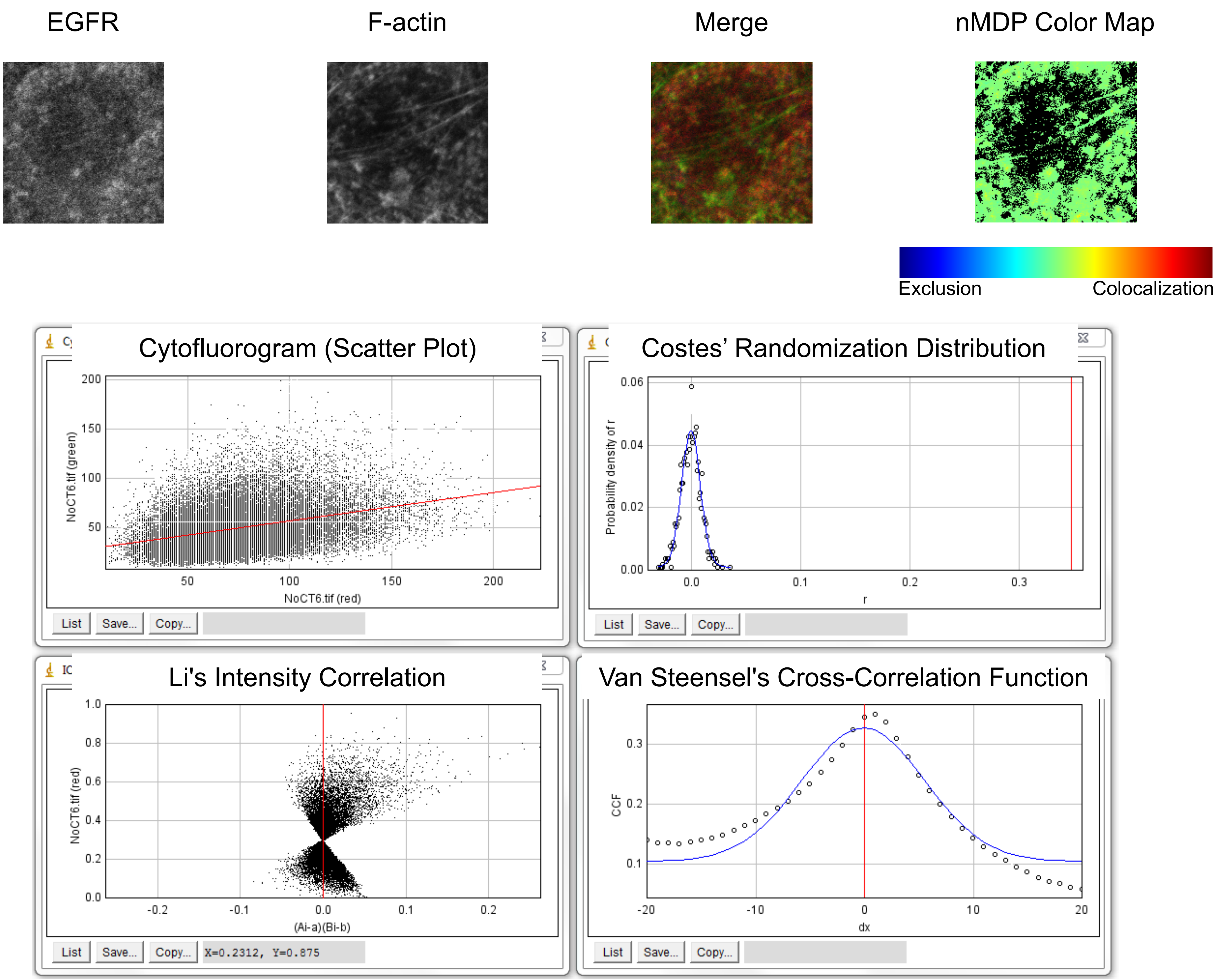

EGFR
F-actin
Merge
nMDP Color Map
Exclusion
Colocalization
Cytofluorogram (Scatter Plot)
NoCT6.tif (green)
NoCT6.tif (red)
Costes' Randomization Distribution
Probability density of r
r
Li's Intensity Correlation
NoCT6.tif (red)
(Ai-a)(Bi-b)
X=0.2312, Y=0.875
Van Steensel's Cross-Correlation Function
CCF
dx
List
Save...
Copy...

Colocalization Parameter	Range	Value	% Colocalization
Pearson's Coefficient	-1:1	0.346	34.6
Icorr	-1:1	0.629	62.9
Manders' EGFR/F-actin<br>Manders' F-actin/EGFR	0:1	0.505<br>0.560	50.5<br>56.0
Li's ICQ	-0.5:0.5	0.125	25.0

Figure S20 (uninfected HeLa cell #3)

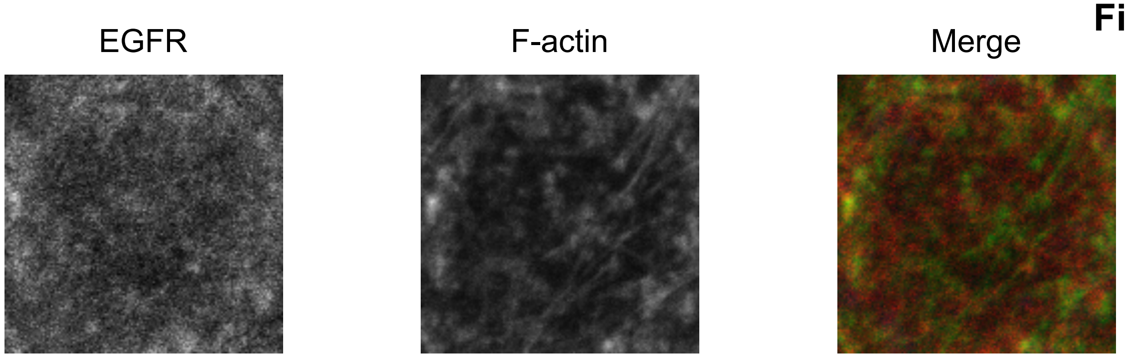

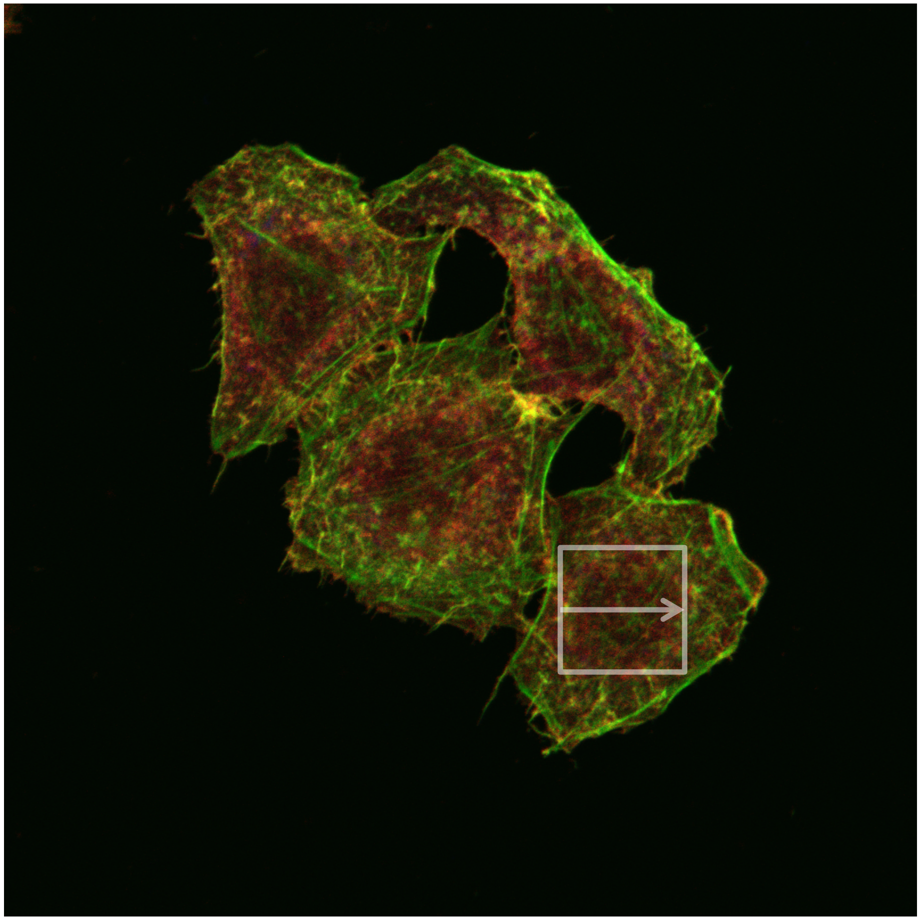

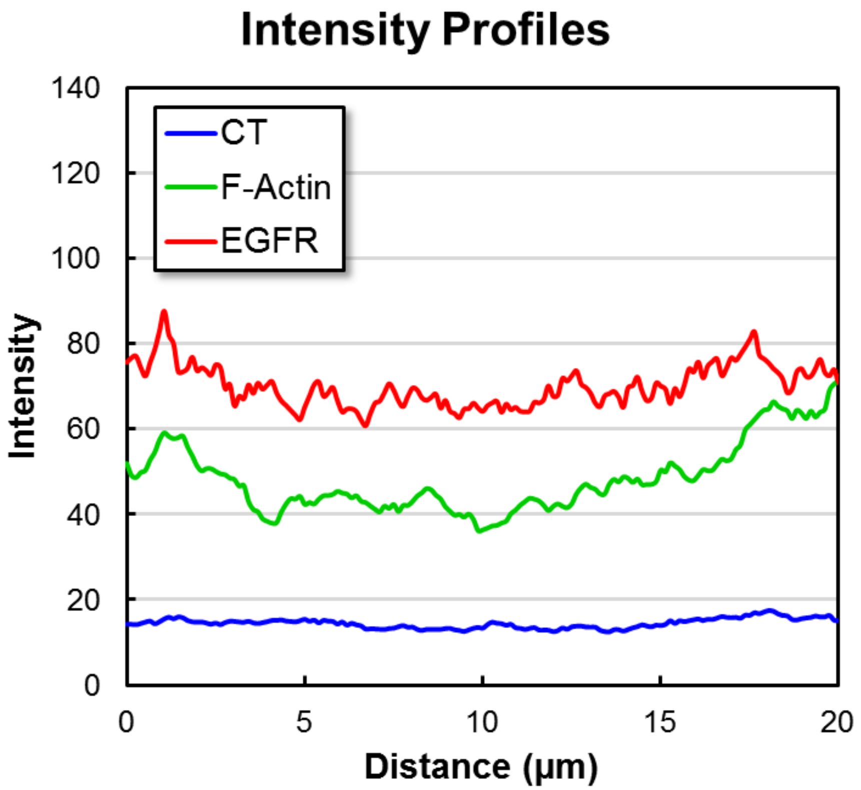

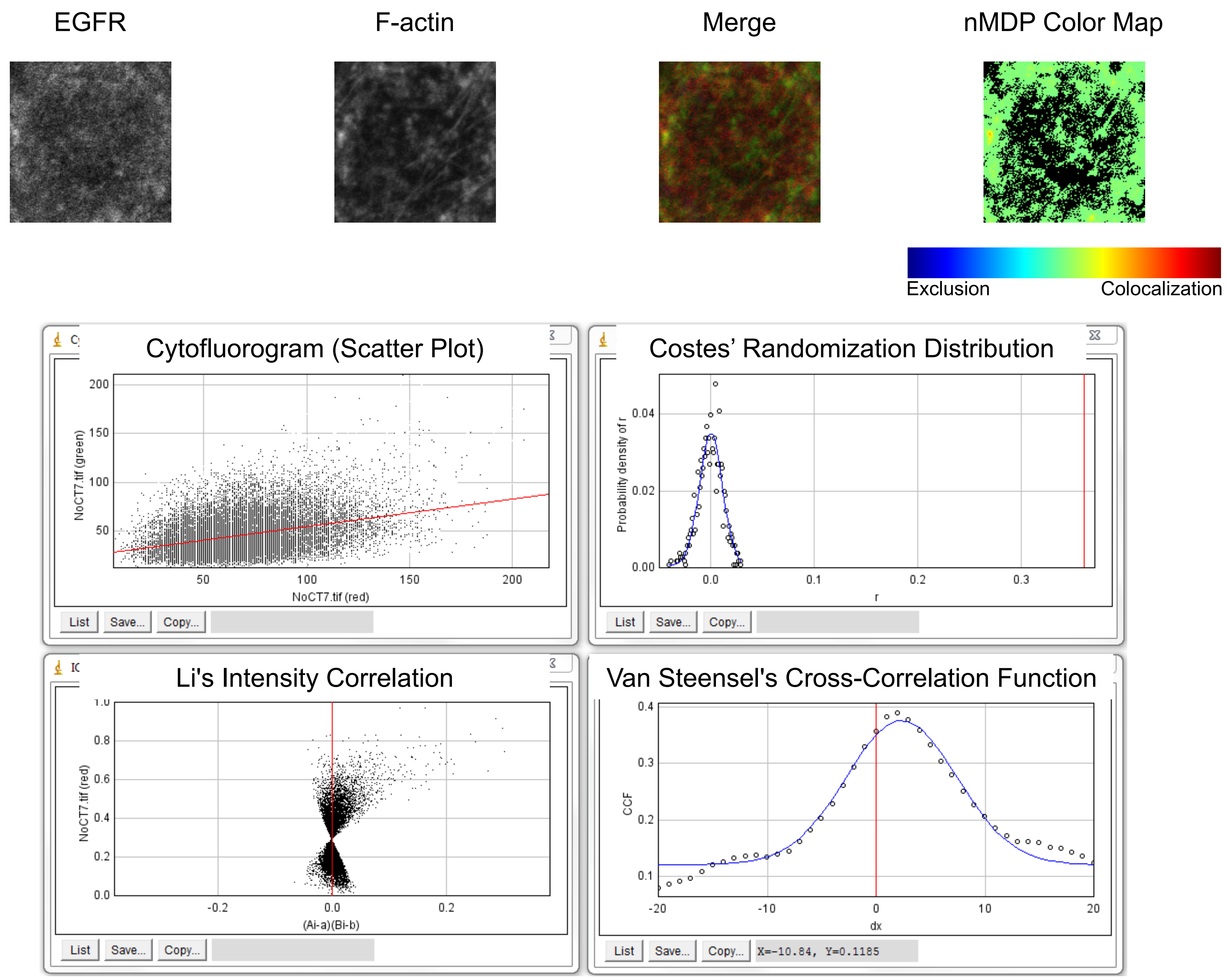
EGFR
F-actin
Merge
nMDP Color Map
Exclusion
Colocalization
Cytofluorogram (Scatter Plot)
NoCT7.tif (green)
NoCT7.tif (red)
Costes' Randomization Distribution
Probability density of r
r
Li's Intensity Correlation
NoCT7.tif (red)
(Ai-a)(Bi-b)
Van Steensel's Cross-Correlation Function
CCF
dx
List
Save...
Copy...
X=-10.84, Y=0.1185

Colocalization Parameter	Range	Value	% Colocalization
Pearson's Coefficient	-1:1	0.358	35.8
Icorr	-1:1	0.623	62.3
Manders' EGFR/F-actin	0:1	0.485	48.5
Manders' F-actin/EGFR		0.569	56.9
Li's ICQ	-0.5:0.5	0.122	24.4
